# Distinguishing out-of-body experiences from lucid dreaming: a phenomenological analysis

**DOI:** 10.3389/fpsyg.2025.1600707

**Published:** 2025-09-15

**Authors:** Marina Weiler, Alexander Moreira-Almeida, Martin M. Monti

**Affiliations:** ^1^Department of Psychiatry and Neurobehavioral Sciences, University of Virginia, Charlottesville, VA, United States; ^2^Research Center in Spirituality and Health (NUPES), Juiz de Fora Federal University, Juiz de Fora, Brazil; ^3^Department of Psychology, University of California, Los Angeles, Los Angeles, CA, United States

**Keywords:** non-ordinary states of consciousness, sleep, phenomenology, altered states of consciousness, consciousness

## Abstract

Despite extensive discussions, a consensus is lacking on whether out-of-body experiences (OBEs) and lucid dreams (LDs) should be considered as distinct categories of experience, and if so, what differentiates them. To address this issue, we first discussed the implications of OBEs and LDs as the same type of phenomenon. We then compiled a comprehensive list of experiential features associated with OBEs and LDs, aiming to identify a distinguishing criterion between them. We conclude that neither the previously proposed features nor the interpretation of the experience can reliably differentiate them. We suggest that disembodiment, or existing without a physical body, should be the key phenomenological feature of OBEs, regardless of other aspects of the experience. Our argument is supported by first-person reports included as supplementary material, indicated by superscript numbers. By focusing on this crucial element, we can better understand and characterize an OBE, distinguishing it from other related experiences.

## Introduction

Out-of-body experiences (OBEs) and lucid dreams (LDs) are experiences that, at first glance, appear to have no relationship with one another. More specifically, the most adopted definition of OBEs includes three phenomenological characteristics: (i) disembodiment (i.e., location of the self outside one’s body); (ii) the impression of seeing the world from an elevated, distanced, egocentric, and extracorporeal perspective; and (iii) the impression of seeing one’s own body from this perspective (Blanke, 2012; Bünning and Blanke, 2005). In contrast, a LD is defined as a dream during which the subject has the awareness that they are dreaming (Baird et al., 2019), i.e., the subject retains their consciousness during the dream and may have control over their actions or the dream’s content.

However, OBEs and LDs present several overlapping aspects, which have sparked discussions among experiencers and researchers regarding whether these experiences should be categorized together. One of the most compelling reasons to consider OBEs and LDs as akin experiences lies in the engagement of high-order cognitive functions during an altered state of consciousness. In both, individuals often question the nature of reality, revealing a shared cognitive aspect underlying these phenomena. Within this context, some authors posit that the resolution to this question hinges on the interpretation provided by the experiencer, suggesting that OBEs and LDs may essentially be two sides of the same coin (Gallo et al., 2023; LaBerge and DeGracia, 2000; Levitan et al., 1999). Simply put, in a LD, the individual becomes cognizant of the dream state, while in an OBE, the individual interprets the encounter as a genuine separation from their physical body (Alvarado, 1997).

This viewpoint is bolstered by research indicating that individuals who have OBEs are more likely to report lucid and flying dreams (Alvarado and Zingrone, 2007; Blackmore, 1986, 1991b), and the cognitive skills associated with both experiences–such as the ability to switch viewpoints–are also similar. Moreover, these experiences share several commonalities: both are often described as involving extremely clear consciousness (with the perception being clearer and more vivid than in ordinary awake experiences), and individuals frequently report that these experiences are profound and life-altering, leading them to strive for more such experiences (Blackmore, 1988). The simplifications, distortions, and additions found in the experienced world can also be similar in both OBEs and LDs, and both experiences involve oddities of lighting and the ability to bring about changes in the environment (Blackmore, 1988). Another significant point of convergence is the occurrence of OBEs during sleep or napping, circumstances in which LDs also occur. Relatedly, it is noteworthy that LDs can lead directly into an OBE (Blackmore, 1991a), and the techniques used to induce LDs and OBEs are remarkably similar.

Nevertheless, this perspective has faced scrutiny from multiple authors who argue that OBEs and LDs are distinct types of experiences. These critics have put forth potential differentiating phenomenological features to underscore the differences between the two phenomena. For example, one proposed criterion is the sensations that precede OBEs, which are considered by some as a hallmark and may be used to differentiate them from LDs (de Foe, 2016; Rogo, 1986; Waggoner, 2008). Another criterion that has been suggested is the level of control the individual has over the environment or their own actions (Gabbard and Twemlow, 1984; Green, 1968b; Stumbrys et al., 2014) or even how vivid and real the experience feels (Gallo et al., 2023; Peterson and Tart, 2013; Twemlow et al., 1982). While no conclusive agreement has been reached, these studies emphasize the difficulty in establishing a reliable criterion (or set of criteria) to define an OBE, and consequently differentiate them from LDs, as suggested by both researchers and experiencers.

This absence of a consensus on the definition of OBEs manifests in various contexts. Some authors rely on the phenomenological experience of seeing the physical body from an elevated perspective (Bünning and Blanke, 2005; Martial et al., 2023), whereas others do not use this specific criterion (Alvarado et al., 1999). In addition to the inherent lack of a clear definition, some authors further compound the issue by integrating their philosophical viewpoints on the nature of reality into the discourse surrounding OBEs. For instance, Crookall (1961) considers OBEs as the actual “externalization” of the mind (see also Osis and McCormick, 1980; Tart, 1998) whereas others assert them as hallucinations stemming from brain dysfunction (e.g., Blanke and Mohr, 2005; Braithwaite et al., 2013; Jalal and Ramachandran, 2017). This lack of a cohesive dialogue among OBE researchers contributes to a situation where studies may inadvertently investigate distinct experiences under the umbrella term of OBEs, or arbitrarily dismiss experiences that could potentially qualify as OBEs.

The lack of consensus on whether OBEs and LDs represent distinct categories of experience and what sets them apart is a matter of significance for researchers in psychology, neuroscience, and consciousness studies (see entries marked as 1, 14, 15, 33, and 34 in the Supplementary material for illustrative examples highlighting the challenges arising from the lack of a distinct and impartial definition of OBE). If they are indeed different, clear distinctions between these phenomena are crucial for accurate research, contributing to a deeper understanding of altered states of consciousness. Furthermore, clarifying distinctions contributes to philosophical and theoretical frameworks, aiding in the development of more accurate models of consciousness and subjective experiences.

With that in mind, our objective was to undertake a thorough exploration of the phenomenology of OBEs and LDs, reexamining the question of what distinguishes them, if anything. Throughout the text, first-person reports indicated by superscript numbers (available in Supplementary material) are included to illustrate and support our arguments. Our proposed criterion builds on previous research (Campillo-Ferrer et al., 2024; Carruthers, 2018). Carruthers (2018) acknowledged that OBEs revolve around the subject’s sense of being separate from their body, though he did not explicitly propose this as a defining criterion for OBEs or as a way to distinguish them from LDs. Campillo-Ferrer et al. (2024) likewise referred to the subjective experience of being located outside one’s physical body. Expanding and refining these earlier proposals, we offer a more comprehensive analysis of the phenomenological evidence and argue that disembodiment–defined as the subjective feeling of existing apart from the physical body–should be considered the key phenomenological feature of OBEs, and the primary criterion distinguishing them from LDs.

Our proposed definition for OBE deliberately steers clear of reliance on any presumed cause, mechanism, or particular interpretation. It is designed to be adaptable for researchers holding divergent views on the nature of reality, refraining from presuming that they constitute the actual externalization of consciousness, and equally avoiding dismissing them as mere hallucinations. Moreover, our model is not limited to specific conditions in which OBEs occur; it encompasses spontaneous OBEs during wakeful states, those occurring in life-threatening situations (i.e., near-death experiences), as well as OBEs induced by psychedelic substances or specific techniques. What lies at the core of our definition is the individual’s perceived relationship to their physical body. While we acknowledge that many experiencers report embodiment in a perceived subtle or “astral” body, what defines the experience as an OBE in our framework is the perceived disconnection from the physical body, not the absence of all bodily form. We aim to offer a definition grounded in rigorous phenomenological analysis and conceptual clarity, explicitly designed to capture the diversity of reported OBE instances across various contexts and conditions, thereby aligning and building upon previous definitional efforts in the field.

## OBEs and LDs as the same type of phenomena

Classifying OBEs and LDs as the same type of phenomenon implies that the only distinguishing factor between them is the interpretation given by the experiencer; in other words, how the experiencer decides to name it. For example, during a LD, the subject may interpret the experience as a dream and “not real,” leading them to behave accordingly within the dream context. Upon waking up, they may continue to view the experience as a product of their imagination or subconscious mind. In contrast, for an experience to be categorized as an OBE, the subject may need to interpret it as occurring in the physical world, with a perceived “real” nature during and after the experience. The subject may believe that they were actually “outside” their physical body and had perceptions from that vantage point. This hypothesis implies that the individual’s belief system and understanding of reality may influence the interpretation and categorization of the experience as either an OBE or a LD.

In the context of interpreting experiences as LDs or OBEs, the specific factors that contribute to one interpretation over the other are still not fully understood and remain an open question for researchers. However, in the field of LD, there have been insights into what makes a person realize that they are dreaming. One proposed explanation is that some LDs are triggered by perceptual inconsistencies or incongruities within the dream. These inconsistencies may involve elements of the dream that do not align with the laws of waking reality, and noticing such discrepancies can lead to the realization that one is in a dream. In an elaborate model, Szymanek (2021) suggests that the transition to lucidity in dreams can occur because of successful reasoning. In this model, the author proposes a Bayesian approach to probabilistic reasoning, where successful reasoning is achieved by the dreamer after noticing bizarre or dreamlike events, or events that have been experienced in previous dreams (but see Campillo-Ferrer et al., 2024, about reviewing literature supporting and opposing the hypothesis). These proposed explanations suggest that the realization of dreaming in the context of LD may involve a combination of perceptual inconsistencies or incongruities within the dream, in which the subject compares the dream plot with the waking reality.

LaBerge (1986) was one of the main authors who challenged the idea that OBEs and LDs are phenomenologically distinct categories of experience. According to him and his colleagues, the difference between the two experiences is not merely a matter of how the subject interprets them, but OBEs may be considered an inferior form of LDs because OBErs may not accurately judge the experience as entirely mental rather than physical, as is commonly believed in OBEs (LaBerge, 1986; LaBerge and DeGracia, 2000). They suggest that OBEs can be considered a form of LD because they are produced by the same mental processes that generate dreams, and they share many of the same phenomenological features as LDs. For example, both OBEs and LDs can be reported during REM sleep (Levitan et al., 1999).

According to their model, the “LD components model,” the difference between LDs and OBEs lies in the “semantic contextual framework,” which operates at the level of declarative knowledge, expectations, and belief, ultimately meaning the belief system used to conceptualize the nature of the experience (LaBerge and DeGracia, 2000). Therefore, LaBerge (1986) argues that OBEs and LDs may not be categorically distinct experiences but rather represent different degrees of lucidity within the same mental state. The authors also suggest that the judgment deficit observed in OBEs could potentially be fixed with practice in LD and learning to recognize false awakenings, sleep paralysis, and other REM-associated phenomena (LaBerge, 1986; Levitan et al., 1999).

However, relying solely on the percipient’s interpretation to distinguish between LDs and OBEs as distinct categories also presents its challenges. As individuals have different expectations, beliefs, and backgrounds, the same experience can be interpreted differently by different people, regardless of the presence of bizarreness and incongruencies with the real world (Alvarado, 1982). In other words, an individual’s interpretation of an experience as either an LD or an OBE may be subjective and can vary depending on their personal beliefs and expectations about the nature of these phenomena. For example, someone who strongly believes in the existence of OBEs may interpret their experience as an OBE, while someone who is more skeptical about such phenomena may interpret it as an LD (Alvarado, 1982). Furthermore, an individual’s interpretation of the same experience may also change over time as their beliefs and expectations evolve. For instance, a person who had previously interpreted a particular experience as an LD may later interpret it as an OBE and vice versa, based on a shift in their beliefs or understanding of these phenomena. Therefore, relying solely on the percipient’s interpretation may not provide a definitive or consistent way to categorize LDs and OBEs as distinct phenomena, as the subjective factors of expectations, beliefs, and background can significantly influence the interpretation of these experiences.

To expand on this idea, let’s consider the example of watching a movie. The movie itself, including its visual and auditory stimuli, constitutes the experience. It is the unfiltered sensory input that we perceive while watching the movie. However, our interpretation of the movie, such as understanding the plot, characters, and themes, is a secondary aspect that arises from our cognitive processing and analysis of the movie. By focusing on the immediate and unfiltered nature of the experience, we can avoid the potential interpretive differences that arise from individual beliefs and expectations. Therefore, we propose that to distinguish between OBEs and LDs, researchers should focus on the immediate and unfiltered sensory experience of the subject during the experience, rather than the cognitive interpretation of the experience that follows. This approach would allow for a more objective and reliable differentiation between the two experiences, while also acknowledging the subjective nature of perception and interpretation.

## Potential distinguishing phenomenological features

Considering OBEs and LDs as distinct phenomena, in turn, implies identifying one or a group of phenomenological features capable of setting them apart. In other words, what features does an experiencer use to classify their experience as an OBE, and what defines a LD as a LD? In this section, we compile a variety of phenomenological features that have been proposed to differentiate these experiences, in search of these criteria.

### Preceding sensations

One of the phenomenological features that has been commonly used to differentiate OBEs from LDs is the immediate body sensations that precede the onset of the experience. Some authors have claimed that OBEs are often preceded by buzzing, energy sensations, vibrations, body paralysis, and sensations of “shooting out” or “rolling out” of the physical body, whereas LDs are rarely heralded by such sensations (de Foe, 2016; Peterson, 2019; Rogo, 1986; Waggoner, 2008; Alvarado and Zingrone, 1998). Relatedly, another attempt to differentiate OBEs from LDs often focuses on how the experience starts or ends, particularly in terms of transitioning to or from the experience (Rogo, 1986). OBErs often describe a conscious transition to the OBE state during the entire process or feeling like they have “fallen out” of their bodies, while LDrs may not be aware of the entire transition and suddenly realize they are in the dream world (Peterson, 2002; Alvarado and Zingrone, 2007). Similarly, accounts of the end of the experience seem to diverge between the two phenomena. OBErs often do not report experiencing waking up after an OBE, but rather a “return” of consciousness to the physical body, sometimes with a noticeable reconnection (de Foe, 2016). On the other hand, LDrs commonly report waking up, having a false awakening, or experiencing the dream imagery “going gray” upon waking (Waggoner, 2008).

However, a closer examination of OBE studies reveals that neither the buzzing and vibration sensations nor the feeling of “leaving” and “returning” to the physical body are universal features of OBEs. Blackmore, for example, found that as few as 12% of OBErs report experiencing shaking and vibration sensations before the OBE (Blackmore, 1984), indicating that the vast majority of experiences do not involve any preceding sensation and, rather, seem to begin instantaneously, with percipients suddenly feeling their consciousness detached from their bodies (Irwin, 1988; Nicholls et al., 2019; Peterson and Tart, 2013). Similarly, 25% of OBErs do not report awareness of a “return” to the physical body (Alvarado, 1997), and nearly 60% of percipients experience a rapid and sudden return (Alvarado and Zingrone, 1998). Likewise, Blackmore reported that 67% of OBErs simply found themselves “out” and “back” again, highlighting the absence of a conscious and smooth transition to or from the experience (Blackmore, 1984; Alvarado et al., 1999).

Additionally, individuals who have LDs often report experiencing unusual bodily sensations such as vibrations, loud humming noises, and the sensation of “rising out” of their bodies and floating above their beds (Baird et al., 2021; Levitan and La Berge, 1991; Levitan et al., 1999). In this context, understanding the physiology of rapid eye movement (REM) sleep has shed light on these sensations and their connection to OBEs. Sleep paralysis, which occurs when a person wakes up from or entering REM sleep, plays a role in this phenomenon. During REM sleep, the brain initiates paralysis of the skeletal muscles (excluding those responsible for eye movement, circulation, and respiration) to prevent individuals from physically acting out their dreams (Hobson et al., 2000). This paralysis is accompanied by a decrease in the brain’s processing of information from the senses, while the cerebral cortex remains highly active (Berger, 2008; Brooks and Peever, 2008). In some cases, the paralysis persists or is active while the person feels subjectively awake, leading to feelings of body paralysis, heaviness in the limbs, and confusion. This occurs due to a lack of synchrony between the part of the brain that prevents the subject from acting out his dreams and the processing of proprioceptive information, leading to feelings of body paralysis or a sensation of melting or increased heaviness in the limbs, and states of confusion (LaBerge, 1986). These hallucinations can manifest as buzzing noises, body vibrations, the perception of people or threatening figures nearby, body distortions, or sensations of electricity within the body (French et al., 2002). Over time, individuals may feel as if they are leaving their physical bodies, either floating upward or sinking through the bed, as their mental body image becomes detached from sensory input from the physical body (LaBerge and Rheingold, 1997).

Based on the available data, it appears that experiencing unusual bodily sensations, such as strange vibrations, loud humming noises, or sensations of electricity within the body, is commonly reported in both LDs and OBEs. Therefore, these bodily sensations cannot be reliably used as criteria to differentiate them.

### Level of consciousness (wakefulness vs. sleep state)

Another commonly cited phenomenological feature used to differentiate OBEs from LDs is the level of consciousness of the subject during the experience, particularly in terms of wakefulness or sleepiness. Some researchers have claimed that while LDs occur exclusively during sleep, OBEs typically occur when the subject is awake (Gabbard and Twemlow, 1984; Nicholls, 2017). There have been reports, for instance, of OBEs occurring during various states of wakefulness, such as childbirth (Bateman et al., 2017), craniotomy (Bos et al., 2016), driving a vehicle, talking, or working (Green, 1968b; Sellers, 2017), among other situations (Baird et al., 2019).

Nonetheless, some have found that only a small percentage (4.5%) of reported OBEs occur during awake states (Green, 1968b), indicating that the majority of OBEs occur during sleep states. Similarly, another study found that 59% of reported OBEs occurred when the subjects were resting, and several subjects claimed that their OBEs occurred during sleep or while dreaming (Blackmore, 1984).

Lucid dreams, on the other hand, were initially believed to only occur during sleep states. It was assumed that if the subject was dreaming, they were asleep. Recent observations, however, suggest that in some cases, the subject can enter a physiological sleep state without losing awareness of their current state of consciousness, directly entering an LD state without any loss of reflective consciousness (LaBerge et al., 1986; LaBerge and Rheingold, 1997). This phenomenon, known as wake-initiated LDs, occurs when the sleeper remains “awake” to the inner worlds of dreams (yet sleeping), giving them the subjective impression of being awake the whole time (LaBerge, 1986). Interestingly, wake-initiated LDs are 4.4 times more likely to be perceived as OBEs compared to dream-initiated LDs (Levitan et al., 1999).

Compounding the issue, it is also possible that although some experiences might appear to start during a state of wakefulness, they are triggered during episodes of “microsleep,” very short, rapid-onset, periods of loss of wakefulness lasting seconds during which individuals typically do not realize they are asleep (Zaky et al., 2021). During microsleep, the brain can be highly active, but the activity patterns across broad regions remain unperturbed by external inputs, such as auditory stimuli (Yong et al., 2019). Importantly, microsleep episodes can occur during continuous visuomotor tasks, such as driving (Poudel et al., 2014), or other tasks that were purportedly happening during OBEs. This suggests that what may be perceived as an OBE could potentially be attributed to microsleep episodes, where the brain briefly enters a sleep state without the individual being fully aware of it. This further blurs the distinction between OBEs and other sleep-related phenomena, highlighting the complexity and multifaceted nature of these experiences.

Taken together, the data above suggests that whether an experience is triggered during a state of wakefulness or sleep is not a criterion capable of distinguishing OBEs from LDs.

### Awareness and lucidity

Undoubtfully, a very important phenomenological feature present in both OBEs and LD is the awareness state that subjects feel during the experience, i.e., the metacognitive ability or insight to ask about the nature of the experience and the recognition that their state of consciousness differs from the ordinary waking state. OBErs, specifically, commonly report a greater than usual degree of mental clarity, and some subjects assert that their intellectual functions were improved in the OBE state (Green, 1968b; Bateman et al., 2017).

Nonetheless, the degree of awareness individuals actually experience during OBEs is not clear. According to some reports, almost 90% of the percipients reported that the course of events during their OBEs seemed quite logical, rather than dream-like (Green, 1968b). According to others, only 44% of OBEs were experienced with wake-like awareness, while nearly 50% of percipients reported dream-like awareness, and 15% compared their experience to a dream or fantasy world (Blackmore, 1984). To reconcile these discrepancies, Peterson and Tart proposed that, similar to everyday life, awareness during an OBE can vary in clarity. Some individuals may report crystal-clear awareness during their OBEs, while others may describe their experiences as muddier or dimmer frames of mind, with different states of awareness (Peterson and Tart, 2013; Berger, 2008). This suggests that the subjective experience of an OBE can differ among individuals and even within the same individual across different OBE episodes.

The ability to reflect on the nature of one’s experience, and whether it is reality or a dream, is not unique to OBEs. Indeed, the presence of a self-reflective state, in which someone is capable of questioning whether an experience is real, sometimes referred to as “pre-LD” (Blackmore, 1984) is necessarily prodromic to being capable of realizing one is in a LD (Voss et al., 2013). It has been proposed that individuals with higher metacognitive abilities during wakefulness are more likely to exhibit metacognition activities during dreaming, and hence have a higher propensity for LDs (Yu and Shen, 2019). This suggests that the level of metacognitive awareness during wakefulness may also influence the occurrence of LDs and the presence of the insight feature in dreams. This has been further supported by research showing a positive relationship between specific meditation practices and lucid dreaming (Gerhardt and Baird, 2024).

However, according to some authors, there is a distinction between LD and OBEs when it comes to the level of metacognitive ability and self-reflective state present in each phenomenon (Irwin, 1988). The argument is that while both LD and OBEs involve some level of metacognitive awareness and self-reflection, it is only in LD that individuals can successfully realize the dream-like nature of the experience. In LD, subjects are believed to have a higher level of lucidity, meaning they are aware that they are dreaming while the dream is occurring. They can objectively reflect on their state of consciousness and recognize that they are in a dream, which allows them to exercise control over the dream narrative. On the other hand, according to Irwin (1988), OBErs typically perceive the exteriorized self as real, similar to how one might perceive a non-LD or an absorbing fantasy. This hypothesis proposes that, unlike in LD, there is no clear realization of the fantasy nature of the experience in OBEs.

To summarize, the available data indicate that both OBEs and LDs can involve elements of awareness and lucidity. As a result, it is challenging to establish clear-cut criteria based solely on awareness or lucidity to distinguish between OBEs and LDs.

### Planning and controlling own acts

The ability to plan and control one’s own actions during the experience is another characteristic that has been proposed as capable of differentiating OBEs from LDs. During an OBE, individuals often report feeling like they are observing their surroundings passively and do not seem to plan on engaging in actions (Blackmore, 1988); on the other hand, during an LD, individuals can often exercise control over the dream’s content, including planning and controlling their actions (Gabbard and Twemlow, 1984; Green, 1968b; Stumbrys et al., 2014).

Nonetheless, a perusal of the literature reveals that a passive observer attitude is not a universal feature of OBEs, with many experiencers reporting actively engaging in tasks and exploring the environment and events as they unfold (Blackmore, 1991a). Similarly, while the ability to perform active planning was objectively verified in LDs (Baird et al., 2021; La Berge et al., 1981), including making voluntary choices, metacognitively checking one’s state of consciousness during the LD, and developing a habit of remembering LD experiences upon waking (LaBerge and DeGracia, 2000), the degree to which this feature is present in LDs can vary greatly (Blackmore, 1991b). One study, for instance, found that in only half of the cases, subjects were able to perform specific pre-planned actions during LDs (Stumbrys et al., 2014), whereas in another the success rate was found to be as low as 14% (Schredl et al., 2018). In this context, Voss et al. introduced the term “lucid control dreams” to differentiate LDs in which subjects can perform volitional acts from those LDs in which subjects do not have the same level of control (Voss et al., 2009).

In conclusion, although it may appear intuitive to consider the ability to plan and control actions as a distinguishing factor between OBEs and LDs, we have presented several reasons why this criterion is not reliable. Therefore, relying solely on the ability to plan and control one’s own actions is not a dependable criterion for distinguishing OBEs from LDs.

### Realism

Another distinguishing feature used to differentiate between OBEs and LDs is the realistic nature of the experience, i.e., how real the experience seems (Gallo et al., 2023); for, OBEs are often described as being “more real than a dream” (Twemlow et al., 1982). It has been proposed that of all features of an OBE, it is probably the reported realism of the experience’s perceptual-like content that most encourages OBErs to interpret the phenomenon as a literal separation from the physical body (Irwin, 1988; Blackmore, 1982). In contrast, LDs are often perceived as having scenery that appears fake or not as real as waking life (Peterson and Tart, 2013).

However, upon closer examination of LD accounts, it becomes evident that realism is also commonly reported in LDs. Many subjects claim that the LD world is often a faithful imitation of waking life or even indistinguishable from it (Green, 1968a; Waggoner, 2008), and that LDs feel experientially real, sometimes described as “more real than real” (LaBerge, 1986). Indeed, the sense of realism, involving the similarity between emotions, thoughts, and events in the dream and wakefulness, is a prominent factor of LDs (Voss, 2015; Voss et al., 2013; Blackmore, 1986). Contrary to the notion that LDs are always composed of unreal content, animals and objects in LDs do not necessarily become personified or start talking, and individuals and objects typically do not change identity as the dream progresses, and the laws of the physical world are usually maintained (Green, 1968a). In LDs, when people appear, they are often characterized and retain their identity throughout the dream. Even if the dreamer is not familiar with them, these characters are often composed of fairly identifiable memories. This suggests that LDs can also involve realistic representations of individuals and familiar environments, rather than solely containing dream-like elements (Blanke, 2012). The presence of identifiable characters and familiar environments in LDs challenges the notion that LDs are limited to incongruent dream-related experiences, further highlighting the complex and diverse nature of dreams and LD experiences (Green, 1968a). It is not uncommon for individuals experiencing LD to acknowledge within the dream that they are dreaming, yet find the dream world to be incredibly realistic (Levitan and La Berge, 1991).

This realistic quality of LD has also been confirmed in laboratory-controlled experiments, where brain activations during dreamed tasks such as singing, counting, and hand movements were found to be equivalent to those observed during actual task performance (Baird et al., 2021; Dresler et al., 2011; LaBerge, 1986). These findings suggest that the inner world of dreams –and LD in particular– can have objective effects on the brain that are no less real than those triggered by corresponding events in waking life.

To conclude this section, we suggest that both OBEs and LDs can be experienced with varying levels of realism, ranging from highly realistic to scenes that appear less real than waking life. Therefore, the degree of realism experienced cannot be used as a criterion to reliably distinguish between OBEs and LDs.

### Environment stability, control, and congruency with the real world

Another phenomenological feature often suggested to differentiate OBEs from LDs is the stability of the mental environment and its congruency with the physical world. In this respect, OBErs typically report encountering an environment that is congruent with the physical world, (Blanke and Mohr, 2005) generally stable over the length of the experience, and less susceptible to change due to expectations or direct manipulation (Peterson, 2019). Conversely, LDrs typically report encountering an environment that can change throughout the experience, sometimes dramatically (de Foe, 2016), and that is subject to willful manipulation (Father “X” A Catholic Monk Print, 1985; Monroe, 1985; Peterson, 2002, 2019).

However, upon closer examination of OBE stories, it becomes apparent that not only do subjects often encounter a mutable environment during their experiences, but the environment may also be incongruent with the real world (Bos et al., 2016). Many OBErs report encountering dream-like features and unreal characters (Braithwaite et al., 2013), which are dissimilar to what one would typically encounter in the physical world (Nicholls et al., 2019). This becomes particularly evident when OBErs claim to travel to remote locations, often referred to as “non-physical realities,” as described in the experiences of Monroe (2014). Monroe (2014) for instance, reported “traveling” to these non-physical realities and encountering human-like beings (Brooks and Peever, 2008), further emphasizing the discrepancies between OBEs and the physical world. These observations highlight that the nature of the environment experienced during OBEs can differ significantly from that of the physical world, including the presence of dream-like elements and encounters with “unreal” characters.

A close review of verbal reports shows that environmental stability and congruency can also occur in LDs (Bünning and Blanke, 2005), with as many as 50% of experiencers claiming they could not shape the environment in any LD at all (e.g., change landscapes/surroundings, let persons/characters appear or disappear), and only 5% reporting they could deliberately shape the environment in all of their LDs (Schredl et al., 2018). In this respect, Waggoner (2008) proposed a more nuanced view of LDs, where much like someone adrift in the sea, the experiencer can focus their attention on a certain course of actions or change in the environment but does not have full control on the success.

Taken together, the evidence above shows that environmental stability, control, and congruency with the physical world can occur, in a graded fashion, in both OBEs and LDs, suggesting this phenomenological feature cannot distinguish between the two experiences.

### Anomalous cognition

OBEs are not necessarily indicative of anomalous cognitive processes, but the alleged ability of OBErs to acquire information without using their physical senses is often used to distinguish OBEs from LDs. Anomalous cognition or experience refers to the ability to acquire information or perceive events through means beyond the known mechanisms of sensory perception (i.e., extrasensory perception) (Cardeña and Alvarado, 2014). During an OBE, some individuals claim to have anomalous cognitive processes, supposedly acquiring information that would be inaccessible to a normal observer in the position of their physical bodies or if they were dreaming. They describe perceiving distant or hidden objects or events, encountering deceased individuals, or gaining knowledge about distant locations (Bünning and Blanke, 2005). Another type of anomalous cognition involves a small number of shared experiences, in which OBErs allegedly meet in the “out-of-body” world or the OBEr is perceived by another person in the physical world during the experience.

While there is some evidence concerning verifiable aspects of OBEs (Osis and McCormick, 1980; Tart, 1998), investigations specifically focusing on the acquisition of information during controlled OBEs are relatively scarce. These limited studies provide inconclusive evidence that individuals during OBEs can obtain distant information. Furthermore, other controlled studies have failed to find positive results (Blackmore, 1982, 1984). As a result, it appears that the ability to acquire reliable distant information should not be considered a consistent feature of OBEs. Additionally, shared OBEs are rare occurrences, and therefore cannot be relied upon to reliably distinguish them from LDs.

While anomalous cognition, in principle, may not occur during a dream in the traditional sense that dreams are stories and images created by our brains while we sleep, there are reports in the LD literature of subjects acquiring information through non-ordinary means while dreaming. For example, Ryback and Sweitzer (1989) report that 66% of surveyed individuals felt they had experienced a precognitive dream, with 8% of them having dream experiences that suggested future-sensing as the most likely explanation. Ryback and Sweitzer’s (1989) subsequent explorations supported his contention that one out of twelve individuals has evidentiary precognitive dreams. These findings suggest that there may be instances where subjects can acquire information through non-ordinary means even during dreams (Cardeña and Alvarado, 2014), blurring the distinction between OBEs and LDs in terms of the potential for anomalous cognition. In addition, shared experiences are also commonly reported among LDrs (Garfield, 1997; Magallón, 1997; Carruthers, 2018). Magallón (1997) describes several different forms of mutual experiences that can occur during dreaming. These include instances where two or more people report having the same or similar persons, places, landscapes, or objects in their dreams, or where two or more subjects encounter each other and even interact in the dream world.

When comparing the presence of anomalous cognitive processes, it becomes evident that they can be observed in both OBEs and LDs. Both experiences allow individuals to engage in actions that defy physical limitations. Consequently, based on the data reported above, it can be concluded that the presence of anomalous cognitive processes alone cannot reliably distinguish OBEs from LDs.

### Sense of time

Researchers have proposed that there may be a difference in the perception of time between OBEs and LDs, which probably relates to how the experience is recollected after the fact. Some authors suggest that the memory of a long OBE experience seems crystal clear and easily recalled in a linear order, while the memory of an equally long LD seems less detailed and more difficult to recall precisely and in order (Waggoner, 2008). This simplistic view of time perception, however, is in clear distinction with other authors who claimed that the sense of time during OBEs falls into three main categories: a sense of time unchanged, a sense of time non-existent (Crookall, 1961), or time passing more slowly than usual (Green, 1968b). For example, one study found that in 60% of spontaneous OBEs, 100% of hypnosis-induced OBEs, and 92% of OBEs occurring during near-death experiences, time was perceived as non-existent (de Foe et al., 2017). Another study found that 45% of hypnosis-induced OBErs claimed to be able to move freely back and forth in time, and 90% of spontaneous OBErs described a very little perception of time (Nicholls et al., 2019). Importantly, this study also found that both hypnosis-induced and spontaneous OBErs reported a clear sense of timelessness or other distortion in their time perception, and none of them reported time consistent with normal day-to-day perception during their OBEs (Nicholls et al., 2019).

Research in the field of LD shows that the perception of time in dreams is similar to that of wakefulness (Erlacher and Schredl, 2004). For example, it has been shown that counting during LD takes a comparable amount of time to perform the same activity during wake (LaBerge, 1986). However, it is important to note that not all LD experiences follow a linear perception of time. Many subjects report losing the sense of time or experiencing time differently in their dreams. Some subjects may report feeling like time is passing quickly, while others may report feeling like they are going back to the past (Dang-Vu et al., 2005). This suggests that the perception of time during LD is not always consistent and may vary among individuals and even within different dream experiences.

These findings highlight that time perception during both OBEs and LDs may vary among individuals and experiences, and it may not be a reliable criterion to differentiate between the two states. Further research is necessary to gain a better understanding of how time is subjectively experienced during these altered states of consciousness.

### Emotional quality and personal transformation

There is significant evidence to suggest that OBEs can have a profound impact on individuals. The emotional quality of these experiences and the subsequent personal transformation that may occur make OBEs highly impactful for many individuals (de Becker, 1968). Research has shown that a significant percentage of individuals who have had an OBE report lasting changes in their lives as a result of the experience. For example, a study found that 55% of subjects who had an OBE reported that their life was changed by the experience (Gabbard et al., 1982). Additionally, 71% of the subjects reported that the OBE was an experience of lasting benefit, and 40% considered it to be the greatest thing that ever happened to them (Gabbard et al., 1982). Moreover, the emotional quality and transformative power of OBEs have been suggested to be unique compared to LDs. Some authors have argued that while LDs may also be impactful, the magnitude of the emotional and transformative effects of OBEs tends to be higher and last longer (Gabbard and Twemlow, 1984). Among some of these OBE-related beneficial effects we can highlight the loss of fear of death, for such experiences convince OBErs that they can exist without a physical body (de Foe, 2016).

However, it is important to note that individual experiences and reactions to OBEs may vary, and not all individuals may have positive or transformative experiences during OBEs (de Foe, 2016). For example, one study found that most OBErs were not sure whether they enjoyed the experience, and were about equally divided as to whether they would like to experience another one; only 10% claimed that it changed their life or beliefs in any way (Blackmore, 1984). In addition, although not common, some subjects reported that their OBE was mentally harmful (Twemlow et al., 1982). While the reasons for such a discrepancy in the OBE-related emotional quality and transformative power are still a matter for future research, they could be associated with the participant’s state during the experience. Indeed, previous work has shown that those who were mentally calm had more detailed and vivid experiences than those who experienced fear at the time of the OBE (Twemlow et al., 1982). Furthermore, in the mentally calm group, the experience was seen as having a more lasting and dramatic impact on life (described as a spiritual or religious experience, an experience of great beauty and lasting benefit, and as effecting a change toward a belief in survival after death) (Twemlow et al., 1982).

High emotional content and subjectively transformative power, however, are not exclusive to OBEs. Indeed, LD has long been known to also have beneficial and therapeutic effects. Since the eighth century, Tibetan Buddhists have placed a special emphasis on LD as a means of achieving self-knowledge, developing a more flexible mindset, and illuminating previously undiscovered facets of the mind (de Becker, 1968; Mota-Rolim et al., 2020). As LDs have been proposed to be a combination between the conscious and the unconscious mind (LaBerge, 1986), LDrs would supposedly gain more conscious awareness of inner concerns while dreaming (Waggoner, 2008). The emotional quality of LDs ranges from a fairly neutral acceptance to varying degrees of excitement, liberation, expansiveness, surprise at the various features of the dream, and possibly appreciation of its beauty (Green, 1968a), which can be ultimately associated with an electrifying sensation of rebirth and the discovery of a new world of experience (LaBerge, 1986; de Foe et al., 2017). This frame of mind allows LDrs to confront otherwise fearful nightmares and anxieties, furthers psychological development toward self-integration and inner harmony by resolving inner conflicts, and fulfills the highest spiritual aspirations (LaBerge, 1986). In addition, research into LD has shown that frequent LDrs possess higher scores on the scales of mental health, assertiveness, autonomy, and self-confidence (Doll et al., 2009), and higher psychological resilience in the face of traumatic stress (Soffer-Dudek et al., 2011).

Collectively, these data suggest that emotional quality and personal transformation cannot effectively distinguish between OBEs and LDs, for both can involve vivid emotions and personal growth or transformation. Therefore, relying solely on emotional quality and personal transformation as criteria for distinguishing OBEs from LDs would be insufficient.

### Seeing the physical body from a third-person perspective

Blanke (2012) proposed that the definition of OBE includes the impression of seeing one’s own body from an elevated, distanced, egocentric, and extracorporeal perspective (Bünning and Blanke, 2005). According to some researchers, this body image would differentiate OBEs from LD, because LDrs have an integrated body image and do not dream of suddenly being in the same room (Gabbard and Twemlow, 1984; Nicholls, 2017).

However, evidence has shown that not always the OBErs find themselves associated with an apparent physical body (Doll et al., 2009). Indeed, one study has shown that only half of the experimenters reported seeing their physical bodies at a distance (Gabbard et al., 1982), whereas another study reported that six of the 15 OBErs did not see their physical bodies (Blackmore, 1982). Another study found that 54% of subjects did not see their own physical body, 44% claimed to have traveled away from their body, and only 24% claimed there was any connection between the OBE self and the physical body (Blackmore, 1984). With similar results, Alvarado found that only 23% of OBErs reported an OBE body similar to the physical one (Alvarado, 1982). Likewise, research has found that in as much as 45% of the hypnosis-induced OBEs and 59% of spontaneously occurring OBEs subjects did not see/perceive their own physical bodies (de Foe et al., 2017). Another study reported similar figures: 40% of hypnosis-induced OBErs stated no contact with their physical body during the OBE, and the majority of the remaining experienced only a partial awareness of it (only one OBEr stated he could feel his physical body); whereas in the spontaneous OBEs, no awareness of the physical body was apparent (Nicholls et al., 2019).

On the other hand, while the perception of one’s physical body is not considered a critical component of the definition of LD, it is commonly reported by LDrs, since they often mention being aware of their physical bodies resting in bed during the experience (LaBerge, 1986; Waggoner, 2008; Dresler et al., 2011). Van Eeden (1913) the original proposer of the term “lucid dream,” described a type of dream that occurs at the very beginning of sleep, during the transition from waking to sleep. In this type of dream, Van Eeden (1913) reported having a nearly complete recollection of daily life, knowing that he is asleep and where he is sleeping. These dreams are usually accompanied by the sensation of floating or flying, while also being aware that the physical body is simultaneously asleep and fatigued (Van Eeden, 1913). Additionally, individuals can have third-person dreams, where the dreamer sees themselves from an external perspective. In these types of dreams, the self is observed and interacts within the dream, as opposed to first-person dreams where the self sees and acts within the dream (e.g., seeing the world from their own eyes) (Dang-Vu et al., 2005; Mota-Rolim et al., 2010). Importantly, although tricky, having third-person dreams differs from having an OBE. While third-person dreams and OBEs involve an external perspective, they are distinct phenomena.

The studies mentioned above provide evidence that the commonly held belief of seeing one’s own body from a third-person perspective during an OBE is not consistently reported in all accounts of OBEs. Therefore, this phenomenon cannot be reliably used as a defining characteristic of OBEs, nor can it be used to differentiate between OBEs and LDs.

## Toward a definition of OBE that can distinguish it from related phenomena

The previous section highlights the challenge of establishing a reliable set of criteria to distinguish between OBEs and LDs, as proposed by researchers and experiencers. Two primary reasons contribute to this difficulty. First, there is a lack of a clear and consistent definition of what precisely constitutes an OBE, making it challenging to differentiate it from LDs. As noted by Metzinger (2005), “At present, it is not clear whether the concept of an OBE possesses one clearly delineated set of necessary and sufficient conditions. The concept of an OBE may in the future turn out to be a cluster concept constituted by a whole range of diverging (possibly overlapping) subsets of phenomenological constraints, each forming a set of sufficient, but not necessary, conditions” (Metzinger, 2005). Second, various authors have proposed different sets of phenomenological features to distinguish OBEs from LDs, with little overlap between them. As a result, arriving at a consensus about the differences between OBEs and LDs is challenging, which is further complicated by the nuanced nature of these experiences.

To enhance clarity on this matter, we propose that the subjective feeling of disembodiment should be regarded as the primary factor distinguishing OBEs from LDs, as the term “out-of-body” inherently implies. The feeling of existing without a physical body should be the key factor in categorizing an experience as an OBE, regardless of other phenomenological features or cognitive interpretations of the experience. Our suggested criterion allows for the classification of a variety of different experiences as OBEs based on the unique subjective feeling of disembodiment, i.e., the feeling of existence without a physical body. For example, an OBEr may suddenly realize they are floating in their living room while watching TV, observe their physical body, and then suddenly “return” to it, while others travel to non-physical realms and have no perception of the physical body. Some may report buzzing and electricity sensations during meditative practice, accompanied by mystical-type experiences characterized by visions of a tunnel and an intense feeling of love and warmth, which may have profound and long-lasting aftereffects, whereas others may have neutral experiences. Some may report willingly “leaving” the body with the intention to acquire information that would be impossible to acquire from the location of their physical bodies and allegedly succeed at it, whereas others do not.

However, the common element in all these phenomenologically complex experiences is that in all of them, the subject feels their consciousness detached from the physical body, and this should be used as a criterion to define an OBE and distinguish them from related phenomena such as LDs. The reason why we selected this feature lies in the fact that OBEs entail the sensation of being separate from one’s physical body, while in LDs the experiencer may be aware that they are dreaming but still feel embodied. LDrs know their bodies are asleep but do not feel detached from them.

In addition, our proposed definition does not take into consideration the interpretation given to the experience, and instead focuses solely on the experiential features, without consideration of the individual’s background, beliefs, or expectations. It is not restricted to specific circumstances in which the experiences occur either: our inclusive approach encompasses spontaneous OBEs, those occurring during life-threatening situations (such as cardiac arrest, disorders of consciousness, and anesthesia), as well as those artificially induced by psychedelic compounds or specific techniques. It also does not determine the nature of the experience, i.e., whether the experience was objectively real or a product of imagination. In this sense, we suggest that even experiences interpreted as dreams should be categorized as OBE if they entail the feeling of disembodiment (Dresler et al., 2012).

By embracing our proposed definition, we can refine our understanding of OBEs and LDs in the following ways:

(i)    Recognizing the complexity of both OBEs and LDs: both OBEs and LDs are intricate experiences that involve a diverse range of phenomenological features. They may encompass various sensory perceptions, emotions, and cognitive processes, and can vary greatly from person to person.
(ii)    Adopting that the key difference between OBEs and LDs is the feeling of disembodiment: OBEs and LDs may share many similarities, but the crucial distinction should lie in the sense of disembodiment. In OBEs, there is a sensation of being separate from one’s physical body, while in LDs, the percipient may be aware that they are dreaming but still feel embodied. In other words, LDrs know their bodies are asleep, but do not feel detached from it. When we learn that the only difference between OBEs and LDs lies in the simple and plain subjective feeling of existence without a physical body (i.e., “I,” “my consciousness” exists without a physical body at this moment), we then understand that both experiences can and have overlapping phenomenological features.
(iii)    Moving away from defining OBEs solely based on the third-person perspective: our proposed definition suggests that OBEs should not be defined by the subject’s ability to perceive their own body from a third-person perspective. This widens our understanding of OBEs beyond this specific aspect and acknowledges that other factors may be at play in defining these experiences. Relatedly, it is crucial to emphasize the distinction between the feeling of disembodiment and the act of seeing one’s own physical body from a third-person perspective. While we can visually perceive our bodies in photographs, movies, and mirrors, for example, it does not imply that we experience a sense of disembodiment in those instances.
(iv)    Eliminating reliance on the subject’s beliefs, backgrounds, and expectations: by embracing the proposed definition, we can move away from relying on the subject’s beliefs, backgrounds, and expectations in categorizing the experience into OBEs or LDs. This allows for a more objective and standardized approach to studying and understanding these experiences, reducing potential biases.
(v)    Reducing confusion in consciousness research: the proposed definition can help minimize confusion in consciousness research by providing a clearer and more comprehensive framework for studying OBEs and LDs. This can facilitate more consistent and rigorous research, leading to a deeper understanding of these phenomena (Erlacher and Schredl, 2004).

We acknowledge that there are limitations to our proposed criterion, particularly concerning the phenomenological features described in this manuscript. A phenomenological feature such as disembodiment, just like lucidity, awareness, and others, are subjective experiences that exist on a spectrum and are not binary in nature. They can manifest in infinite shades of gray between black and white. For instance, some subjects may report detachment from specific body parts, while others may somewhat sense full-body detachment. However, among the phenomenological features described above, the feeling of existence apart from the physical body is relatively easier to recognize, and if a subject is in doubt, it should not be considered a disembodiment. This can serve as a relatively more reliable criterion in differentiating OBEs from LDs, as it indicates a distinct sense of separation from the physical body. Nevertheless, we acknowledge that even this criterion may have limitations and may not be foolproof in all cases.

Furthermore, we acknowledge that OBEs and LDs may exhibit additional distinctions, given the potential differences in their respective brain signatures (Rogo, 1985). However, while some progress has been made particularly in the field of LD neurophysiology (Baird et al., 2019; Dresler et al., 2012; Erlacher and Schredl, 2004; Erlacher et al., 2013; Stumbrys et al., 2014; Voss et al., 2009) there is still insufficient knowledge for a comprehensive comparison between the two experiences. Additional research on the neural correlates of OBEs, in particular, will be necessary to further enhance our understanding of the distinct brain signatures underlying these phenomena.

## Conclusion and recommendations

The unsuccessful quest to establish differences between OBEs and LDs relates to two main reasons, namely the lack of a proposed phenomenological characteristic that ties all OBEs together, and the lack of a consensus regarding the divergent criteria between OBEs from LDs. To address these issues and make improvements in the field, we need to identify which aspects can be generalized among OBEs, so that it would provide the foundation from which OBEs could be distinguished from LDs. While some authors have proposed specific phenomenological features as potential differentiators, we argue that no single feature or set of features that have been proposed can reliably distinguish OBEs from LDs. Additionally, the approach of interpreting and making sense of the experiences also has its limitations and challenges as a potential differentiator.

We propose that the subjective feeling of being detached from the physical body should be the key factor in categorizing an experience as an OBE or LD, regardless of other phenomenological features or interpretations that accompany them. When we understand that the only difference between OBEs and LDs lies in the simple (yet comprehensive) subjective feeling of existence without a physical body–as the name “out-of-body” implies–we realize that both experiences can have overlapping phenomenological features. Therefore, we suggest that even experiences interpreted as LDs should be categorized as OBEs if they entail the feeling of disembodiment.

Our proposed criterion allows for the classification of a variety of different experiences as OBEs based on the unique subjective feeling a person may have–i.e., the existence without a physical body–, and it differentiates them from LDs. By focusing on the subjective feeling of disembodiment, we aim to provide a clearer and more reliable distinction between OBEs and LDs, which may aid in future research and understanding of these phenomena.

Our proposed definition of OBE and subsequent criterion to distinguish it from related phenomena also have implications for virtual reality experiments. In some of such experiments, participants may report a sense of detachment from their physical bodies due to the immersive nature of virtual reality technology. However, unlike in a genuine OBE, these participants may not experience a complete detachment from their physical bodies in the sense of existing without a body (Martial et al., 2023). Instead, they maintain a connection to an avatar or virtual representation, which keeps them embodied in a virtual form. By defining OBE and establishing this distinguishing criterion, we can ensure that researchers studying phenomena related to OBE in virtual reality experiments can differentiate between genuine OBE experiences and other immersive virtual experiences that may resemble an OBE to some extent.

Finally, we offer three recommendations for researchers studying OBEs and LDs:

(i)    Focus on the detachment feeling: when assessing whether a subject experienced an OBE or an LD, prioritize asking questions about the subjective feeling of detachment from the physical body. This should be a key factor in differentiating between the two experiences. If the subject reports a sense of disembodiment, in the sense of existing without a physical body, then the experience should be classified as an OBE, regardless of other phenomenological features reported. On the other hand, if the subject reports knowing that the body rests, or seeing the body in a third-person perspective, but does not feel detached from it, it should not be considered an OBE.
(ii)    Be cautious with phenomenological features: phenomenological features commonly associated with OBEs, such as flying, floating, vividness, and anomalous cognition, should not be solely relied upon to define OBEs or distinguish them from LDs. Even if the subject reports these features, if there is no accompanying sense of detachment from the physical body, the experience should not be classified as an OBE.
(iii)    Minimize interpretation based on beliefs and expectations: researchers should be mindful of the possible influence of a subject’s beliefs, background, and expectations on their interpretation of the experience. Avoid relying on subjective interpretations provided by the subject, as these may be biased.

By following these refined recommendations, researchers can enhance the rigor and accuracy of their investigations into OBEs and LDs and contribute to a better understanding of these intriguing phenomena.
