# Supplementary material for: Distinguishing out-of-body experiences from lucid dreaming: a phenomenological analysis
Source: Front Psychol. 2025 Sep 15;16:1600707. doi: 10.3389/fpsyg.2025.1600707 (PMC12477129; doi:10.3389/fpsyg.2025.1600707)
Supplement: Supplementary file 1 [file Supplementary_file_1.docx]

# ^1^ Below are some examples of stories that describe sensations that may precede OBEs:

I now identified [these sensations] as a pre-OBE state, a transition state between the physical reality waking state and the full out-of-body state. In this transition state **I feel fully awake but find I cannot move – my physical body has become paralyzed. I can see and hear, but although it seems like I do this physically, I sometimes see and hear things not physically present. And although I can see, my physical eyelids remain shut. For example, I see a room that looks like my physical bedroom, but I may also see other entities in it – from ‘ghosts’, to ‘angels’, to ‘aliens’**. Many others have reported experiences like these during sleep paralysis. In my early experiences in this pre-OBE state, **I often felt waves of energy rushing up and down my body and heard a buzzing vibration** **sound**. Experientially my consciousness had dissociated from my physical body and associated with a second non-physical body, but the second body in which I found myself often still seemed attached to the physical. If I could intentionally speed up the vibration/wave, my second non-physical body became unstuck, and I could move away while the physical body remained in place, by ‘rolling out’ of it, or by floating out of it. During all of this I felt fully awake in a similar way to that of when physically awake. (de Foe 2016)

A friend of mine came to pick my brother and me up at my dad's house. My brother and I went to the car, and I got in the back seat. My brother realized that he'd left something in the car, and he went to get it. He didn't say what it was, he just ran into the house to get it. I figured my dad would stop him to talk to him and that he would be a few minutes, so I laid my head back and relaxed. My dad had introduced my brother and me to remote viewing a few days earlier, and I was fascinated by it, so once I got comfortable in the back seat of the car, I decided to try to see what my brother was doing in the house. Suddenly, **I heard loud buzzing and crackling sounds, and they started getting louder. At the same time, I could see a bright light coming from out of the distance (I had my eyes closed). Once the light and sounds reached a climax, I heard a loud bang like something being ripped apart. I guess the best comparison would be a sonic boom.** Immediately after that, I could see everything around me in the car, and I began to float above my body and out of the roof of the car. I hovered about 3 feet above the car for a few second, long enough to see my brother walking out of the house, carrying his CD's in a carrying case. If I had been watching from inside the car, I wouldn't have been able to see the CD's. Then, my brother got back in the car and shut the door. I came back to my body slowly and opened my eyes. (OBERF)

# ^2^ One of the phenomenological features commonly associated with OBEs is the ability to consciously transition to and from the experience. This feature has been used by experimenters and authors as a distinguishing criterion between OBEs and LDs:

The only essential difference between [my OBEs] and my lucid dreams is that **I am totally conscious when I enter this other state of consciousness** [OBEs], whereas my lucid dreams always begin with a non-lucid dream and then it becomes lucid. (Father "X" 1985).

[...] My first problem was...to get away from the physical body that usually claims some sort of hold on my astral counterpart. This time I had no problem: **I turned over so that I was face down (my body was face up). Then I pushed myself up until I was on my hands and knees. My hands, knees, and legs were still in coincidence with the physical body. Then I just withdrew my limbs one by one, and climbed off the bed**. I was free from the body. (Peterson and Tart 2013)

# ^3^ The following examples show a lack of smooth transition to or from the OBE, in which the subjects suddenly found themselves “out”:

About twenty three years ago, during a troubled period in my life, I had a vision of my own death. I was awake but in a meditative, relaxed state whilst sitting on the floor, listening to music. This is what happened. I was very old, over 90, and saw myself close to where I was brought up, on the south coast of England. I was standing on the cliff tops looking out to sea. This was something I have done hundreds of times and it felt very familiar to me. **Suddenly, I stepped out of my body and saw it fall to one side, just like I had removed an old coat or jacket I no longer needed**. I had no attachment to it and I was surprised to find myself looking at my body because I didn't know I was about to experience my death. I was not in the least alarmed or anxious. I felt at peace. Just then, I unfurled the most amazing wings: pristine, white and very strong. They had been furled for a long time but were just ready at that moment, not a second too soon or a second too late. I set off towards the sun, marveling at the beauty of the ocean below me. It was azure blue with diamonds of light twinkling up at me. The sun's heat and light drew me towards it and at the same time, I was willing myself towards it. I knew with absolute certainty that the golden light was where I belonged. I was going home and I was SO excited to get there. At that point, the vision stopped. After the vision, for the next three days I was in a strange state. I remember going to the supermarket, looking at all these people and trying to sense the spirits behind the bodies. I remember thinking 'It doesn't matter what our jackets are like: old, young, black, white. We are all spirits wearing body jackets and one day, when we are ready, we will go home'. (OBERF)

After reading the book "Three Magic Words" by U.S. Anderson, I did as the book suggested and meditated on a daily basis following the guided meditations in the book. After about a month of meditating, one morning as **I was laying in bed about to get up I was suddenly found myself in a soft light**. I felt that I was in the light, that the light was pure love. The feeling of total love is impossible to explain, it was as though the light was God, and that I was as one with the light. I remember being told something very profound yet very simple. Like that was the answer to everything. It was not told to me by a voice, rather by telepathy. However after leaving the light and returning to my body I could not remember what I was told. [sic] (OBERF)

**Another one of my experience's happened when I just woke up out of a dream**. I was laying in bed and like usual the first thing I did is go to turn on my lamp in my room. When I went to flick the switch nothing had happened which made me think I had a power cut but I soon realized that I was floating a small bit out of my bed. So I straight away headed for my door to see how far I can get without going back into my body. As soon as I got to my door I saw only what I can describe as a camera flash go off behind me but because being out of your body you learn to expect that anything can happen. I ignored it and went into my hallway. Soon as I got halfway down it everything around me started to move at what seemed 1000 mph. The sheer speed of everything was immense. I started to see children's toys on the floor in the hallway and the doors changed position and shape and even the hallway got longer. But shamefully I got too scared of this surprise traveling and I managed to get myself back to my body before I even looked around this new place. [sic] (OBERF)

# ^4^ Below are some examples of stories that describe sensations that precede LD:

I wake up in the middle of the night, to the sound of my own voice set as my alarm clock. “Stay still, don't move, remember, remember remember, everything that you need to remember.” The alarm then auto-dismisses as I start to process my thoughts and REMEMBER my goal to do (W.I.L.D) lucid dreaming. I use my awareness to scan my body. **I am on my right side and want to roll over badly. I ignore it then an itch starts in my shoulder, I ignore it. Both impulses get stronger – Ugh! - But I ignore them. It freeking hurts. I ignore it, the itch and pain then both vanish. I feel funny; a feeling that one might say is heavy as lead and one might say is as light as feather. Light vibrations start like electricity, tickling up my spine and** **down my fingers and I feel numb. The vibrations get even stronger and I hear a HMmmmmmmmmm sound, buzzing in my head and scarry whispers...FIGHT OR FLIGHT FEAR RESPONSE SETS IN. I feel it in my belly like I'm about to drop off a roller coaster.** I think to myself, “Don't let this fear snowball. Balance emotions, no fear, no hate, no anger, no sorrow, only feelings of love, joy, excitement, and respect.” I start to visualize my room around me with as much detail as I possibly can. I start to visualize what it would look like to make a rolling motion out of my bed onto the floor of my room. I then put all of my attention onto my left shoulder blade area, then my right shoulder blade area, and rotate my awareness back and forth back and forth, until a rocking sensation starts. I set a strong mindset, with high confidence, no doubt, truest self and know it will happen. The words (ROLL OUT) are going over and over in my head. The magic happens and I roll out onto the floor of my room. The vibrations stop. It's dark and I can feel my knees on my carpet. With my hands, I touch my surroundings. I can feel everything, but I can not see. It's gloomy like a foggy darkness of gray and black surrounds me. I bring my attention to my hands then start rubbing them together putting them right in front of my field of vision. I can start to see them appear and I yell out, “Vividness now!” “Clarity now!” and “High definition vision now!” all while visualizing my room around me with as much detail as I can. My vision shifts like a light has come on and I can see. It's still not very clear, but I can make out my room. I then start touching everything around me, my body, my dresser, the carpet, the bed as I am paying close attention to the detail and textures. My environment becomes much more clear and vivid. I stand up and try to float up into the air by jumping and flapping my arms like butterfly wings. It serves as a reality check before I jump out of my window! I run, jump, and smash right through a glass window like a crazy man and land in my dad's garden. It's dark out but I can see well, and the stars look amazing. I lay on my back and look up at them, amazed at how clear and close they are. My vision changes like a telescope and I no longer have a sense of a dream body. I am in a visionary state! I enjoy taking in the vast beauty of the stars, other worlds and galaxies for about one minute. It is amazing. Then . . .total darkness. From this darkness my sense of a dream body comes back. I can feel my knees on a hard surface. I stand up and begin rubbing my hands in front of my face. I can see them and then I look around. There is a big concrete-like square table or slab in the middle of a room surrounded by five pillars. In between each pillar is a torch on a metallic metal pole and there is a man standing off to the side. He wears gray robes and does not move or even acknowledge my presence. Then I see her - the lady in red. She is beyond stunning - like you might imagine a goddess would look. She wears beautiful ruby-red jewelry and an outfit of all red that I can't even find the words for. She is a total knock out, beautiful. I tell her I am lucid and she laughs at me and says nothing. A door opens up behind the pillars, and she makes a “follow me” waving gesture, then turns around and begins walking away. I start to follow her down a rather dark and gloomy hallway that is also lit up by torches on metal pillars of some sort. Then . . . “Meow, meow, meow.” My cat Gismoe wakes me up, rubbing his face against mine. “Meow, meow, meow.” The whole right side of my body is SORE from ignoring the earlier roll over signal! I think to myself, “WTF, cat? I was totally about to get lucky!” (LDE)

After a failed WILD attempt – **the vibrations wake me up** – I manage to stand up within a dream, fully conscious. I am in a room that is not mine. I move out into a dark hallway with beautiful colors in the background and, without having it planned beforehand, I ask aloud to ‘the awareness behind the dream’ “How can I work the limits?” On the hallway wall I see some letters written in light, something like ‘galata’ in Greek, but they change and I cannot read them well. It seems that the dream is getting blurred, but I move fast and I recover it. While I keep asking the question, I reach the shuttered door of a balcony, I raise the shutter – the noise is identical to the one of my childhood home! – and I just jump off the balcony without any thinking. Enjoying the fall, I lose consciousness and wake up. (LDE)

# ^5^ There are examples of OBEs occurring during awake states:

A typical morning at 8 am on an almost-completed construction site. I was asked to go around and check the lamp post bases. My first base was on the opposite side of a large car park. As l proceeded to walk over, the vehicles passing on the main road in front of me seemed to stop. Then shortly after stopping they would gradually increase. At the same time, l could hear this voice saying, 'It's your last chance.' I was still consciously aware of my surroundings as **l was still walking to the chosen lamp post**. The message, 'This is your last chance' also became more repetitive. When l arrived at the lamp post base l began my intended work. From nowhere, a young girl with plaited hair and a long nightdress (Victorian looking) appeared and was smelling of the lily of the valley. I could tell she was friendly. She held out her hand, which l took. l was approximately 8 to 10 feet off the ground and looking down. l could see myself motionless […] [sic] (OBERF)

After my water broke, the contractions became mind altering. I was screaming for hours. That I remember. But I had said no medication. Even if I asked for it, I had refused it for myself. I had no idea what labor felt like and I had now idea I was having back labor (when the head comes out face up instead of face down so the skull drags along the spine the whole way down). **At some point closer to the delivery I realized I was on the ceiling of the hospital room watching everyone**. I could see my husband, the midwife, the nurses coming in and out. I could see myself lying in the bed, all the monitors etc. Time stood still and all of a sudden I was back in my body and I had the INCREDIBLE urge to push. Everything after that was about the trauma of the pain. For months I would cry just talking about it. I definitely suffered PTSD and I believe the pain was so excruciating, I had an out of body experience to survive the experience. [sic] (OBERF)

As a teenager **I often used to put on my favorite music while alone in my room and danced to it**. My eyes were closed and **I enjoyed the music and the dancing**. A feeling arose that I was more than my body, I was larger than it and was filling up the room. It was kind of puzzling because I saw myself as if looking through a telescope the wrong way round - my body (as other things in the room) seemed very very small, and very far away, while I was very very large. There were no thoughts, no emotions in me. I liked the experience very much and was in awe. It was a very peaceful, relaxed, and serene feeling. It was just 'I AM'. [sic] (OBERF)

**I was driving through a train underpass**, the road goes down underneath the train tracks. Two lanes going this way and two lanes going that way, with a big concrete divider in the middle. The lanes were narrow and it always frightened me to drive through it especially if there was a car along side me. I always took the lane next to the divider, drove close to the divider to make room for any car in the other lane. I always used full concentration when driving through there because it was scary. I was born in Calgary and drove through this thing on a regular basis. Then one day I was about to drive through there while paying no attention at all, when I realized this I got very frightened, then I was out of body about 30' in the air behind the car viewing the distance between the car and the concrete divider. I watched as the car went through and was steering the car from out of body as it went through, when ever I moved the steering wheel I was briefly back in the car to watch how much I moved the steering wheel, then out of body again to watch the car go through. Soon as I got by the concrete divider I was back in my body. I did not freak out as this happened, just go with the flow. All the correct sounds were in place, while out of body I could hear the car going through and other extraneous sounds from the out of body position, and when I flicked back behind the wheel all the regular sounds from being behind the wheel were there, didn't miss a thing. I drove through perfectly, not a flinch. I was so freaked out afterwards I had to stop for coffee at the truck stop just up the road. That was the last time I ever drove through there inattentively!!! [sic] (OBERF)

I was 4 years old. My grand mother was burning leaves in the yard, we lived in the country. I was running around picking stuff up and throwing it into the fire. I went to the area where the barrel is that we burn trash in. There was an aerosol can that had fallen out of the barrel. I picked it up and threw it into the fire. A few seconds later the can exploded. The shrapnel went flying and some of it hit me. The pain was amazing and the sound of the explosion was deafening. **The next thing I know, I'm standing next to a tree watching a little girl run past me screaming. My grandmother said I was running and screaming**. During the oobe I didn't feel the pain of the burns, and I didn't feel scared. After I came back to myself, the pain was incredible. I still have scars on my arm today to remind me of that day. [sic] (OBERF)

# ^6^ OBErs often report a greater than usual degree of mental clarity and awareness:

About 6 times in two months, when in bed trying to sleep, felt my body suddenly paralyzed and a strong frightening noise in my ears. Tried to move and wake my partner besides me but completely unable. Then a loud "pop" sound, feeling like flesh ripped of my body and suddenly legs floating, like pulled up by something and then rest of body out. Each time find myself exactly room in front of the bed where I lay down (sometimes my own, sometimes my daughter's, sometimes the sofa). Seeing each time the furniture and objects around like lit from inside (by themselves) I felt everything had its own light and sound as well. Tried to move, just thinking about going down the corridor and then being "sucked" to the place at an amazing speed. Feelings of lightness, **I could think incredibly clearly, everything seemed much more real than reality.** Sort of concepts or information about life and its purpose coming to my mind. Everything around recognizable but with funny distortions. Sometimes some object or shape I wouldn't recognize. I could not see clearly my body lying down but could feel the shape somewhere there. I could perceive though the body of my partner bed. Then suddenly back and sit up in my bed sweating, not understanding what happened, quite frightened to go back to sleep for some days. It's happened about six seven times since then and not so frightening anymore though I can't control the process voluntarily. Also find that depending on my previous emotional state or types of thought the experience can be pleasing or terrifying. Most striking the quality and clarity of consciousness and the vividness of feeling. When it happens it seems as "this reality" is a dream. And you don't find important the things that you usually care about during the day. [sic] (OBERF)

I am resting my head on the window of an airplane. The passengers on the plane are quiet and the plane is quite high in the atmosphere. The only sounds are the mechanical humming of the plane and the occasional shifting of passengers. Its a beautiful clear day. We are up where the air is thin and the sky is light light blue . I look downward to earth onto white fluffy clouds and an increasingly darker blue sky below me. My feeling is that of a child in the backseat of a peaceful car ride, detached from worldly problems, not having to do anything. I close my eyes to rest them. **Without any warning or conscious effort (never having experienced this before) and for no reason that I consciously am aware of still today**, I find myself slipping out of my body from the top of my head. And instantly find myself floating and flying (moving) through a place (field? dimension?) of pure potential energy. The qualities of it are as follows. It is exquisitely perfectly dark, no light what so ever is emanating from anywhere. It is infinitely exquisitely silent. No sound emanates from anywhere. It is infinitely vast. It does not have a beginning or end but does have a 3d spatial quality. It is perfectly peaceful in every way. It is so still, harmonized and GOOD. My first though is something like. 'Wow, the infinite is so easy to understand!' But there is more to it. It seems very very alive. Next I turn my attention to myself. I am whole, intact, conscious, and I recognize myself. I have a boundary that contains myself and it seems to be organized in the shape of a body. But it was non material . Almost as if it were an outline or an energetic field in the shape of a body. I seem to be moving horizontally similar to how superman might fly without his arms extended. I seem to be moving forward. These things don't seem to grab my interest particularly. I think about it for a quick moment and turn my awareness back to the dimension I find myself in. What seems exquisite about this space is the PROFOUND POTENTIAL ENERGY. I am going to be direct here because it was simply a fact. It was what it was and it was quite obvious. The darkness was in fact light particles without any force acting upon them. Since the space was infinite there was room for everything to be at rest. The light was not extending. It wasn't collapsed light particles, it was more like still light where the light spectrum encapsulated itself. An endless amount of light 'seeds' or 'individual light densities' that if acted upon with force could create images, sounds, matter. It was white light (or spectral light at rest) Each particle of nonmoving light had such profound potential energy and as if it 'waiting for a creative force to act upon it'. An infinite amount of these dense light particles extended and nested amongst each other creating this field, that I am flying in. A field with no forces of motion acting upon it because there was infinite space for everything in the universe. No cause and effect. It was humming without sound, vibrating without movement, yearning with this potential power. It seemed to be yearning to be birthed . It was truly alive but not activated. Pure potential energy. All this was so obvious. The Void is anything but empty. It is all the potential energy in the universe. [sic] (OBERF)

# ^7^ During an OBE, similar to in waking life, consciousness can vary in clarity from crystal clear to muddy. Subjects have also reported experiencing dimmer frames of mind and altered states of awareness during OBEs:

Usually, my consciousness is completely normal in all respects. At times my consciousness is very strong, and I feel more awake and aware than in normal life. **But sometimes my consciousness feels very weak**. I've often used the analogy between consciousness and a light bulb that is on a dimmer switch. (Peterson and Tart 2013)

I've had OBEs where my consciousness was more intense than normal waking life. **I've also had OBEs where my thinking was more "muddy"**. (Peterson and Tart 2013)

[…] Then something happened that might be difficult to describe. **It seems like my consciousness dimmed to the point where I couldn't organize memories.** The memory I have is of time standing still. I remember tiny flashbacks of things happening, but they all seemed to have happened at once. I remember seeing DB, and I remember him walking back and forth. […] **But my consciousness was so dim that I didn't have conscious control, and my memory is so jumbled about that part that I can't say any more** [for certain]. (Peterson and Tart 2013)

**I am not completely aware of what happened**. All I know is that while I had a dream about my husband I left my body and visited him in bed. Due to my husband's snoring I sleep with our daughter. He tells me that this is not the first time I've visited, but this was the strongest visit, since he actually saw me. While I was having a dream about him (totally unrelated to what he experienced), he felt a hand on his shoulder. It was a strong grip, he could not move his hand. I then began making smooching (kissing) sounds. I often do that (while awake), from across the room over the phone, giving him kisses even though I can not reach him. I hope this makes sense to you. After a few seconds he finally smooched back, I let go and began to leave. He rolled over to see me disappearing out of the room. **I don't remember doing this at all.** I woke up from my dream, having to go to the bathroom. [sic] (OBERF)

# ^8^ The following is an example of a “pre-LD”, where an individual is not yet aware of being in a dream:

On one occasion in these circumstances, I dreamt that my daughter-in-law was near me. I spoke to her: Louise, I said, look, I am seeing clearly, but I am afraid it is only a dream. **Am I really awake?** pinch my arm so that I can be sure of it. She did not answer, but pinched my arm so could hardly feel the pressure of her hand. ‘Harder’ I said to her. She obeyed, but no doubt from fear of hurting me, pinched me so gently that I could only just feel it. However, the test seemed to me conclusive; and, to tell the truth, **I was so convinced of being awake** that I spoke to her less to convince myself than to convince her. **Not for a moment did it enter my head to think that if I was dreaming**, this verification would prove nothing since it would itself be part of the dream. So I was convinced and very happy. (Green 1968)

# ^9^ A passive observer attitude claimed to be typical of OBEs can be exemplified in the following report:

On the 1st occasion I thought, ‘This is strange. What is happening to me?’ and then I just watched and listened to what was happening around ‘me’.[…] Another time I am sitting in a chair and seem to float out and just stare at myself. (Green 1968)

I disembarked from a 3 hour flight in the afternoon and was walking down towards the baggage area when I saw my daughter waiting to greet me. As soon as I saw her, suddenly I was somewhere above watching me walking down to greet my daughter. **I was in the scene yet watching it**. It lasted about 5 seconds and ended when we met each other. No sounds. No light. **Just complete silence as I watched**. [sic] (OBERF)

I had been feeling inexplicably tired all day. I had not done anything that was outside my normal routine, because of feeling so tired for no apparent reason I had been having a pretty lazy day. I decided to go lay in bed for a little while to rest. After a little while of relaxing I started to feel as if I was rising through the air, **and then could see my bedroom from a new perspective as if I was floating above my bed. I was in the air looking down on myself lying there. As I watched I saw a pink/red orb emerge out of my ear and to a few feet away from my head.** After a few seconds the orb slowly moved back into my head, then I was suddenly back in my body again. There was nothing unusual building up to this event and I have never had an experience like this before. I felt quite disturbed but very intrigued by my experience however have no idea what it actually means or how it happened. After I felt at peace and very mellow, as if I had been meditating for a long time but also rejuvenated and well rested. Almost as if my out of body experience had healed me and refreshed me in some way. I still do not understand what happened or why. [sic] (OBERF)

# ^10^ The following stories show that OBErs also assume an active attitude during their experiences:

I felt excited and surprised at first, and it amused me that I could look down on myself. Then I had an urge to leave the room and see what my mother was doing, or my brother, or my sister. […] I remember thinking I was anxious to make the most of the opportunity. In a flash my mind was made up. I would go into the street and ask the advice of the first man I met. (case 52) (Crookall 1966)

After reading a book, "Science of Self Suggestion and Self Hypnosis", I decided to try the techniques outlined. After lying flat on my back and attaining a totally relaxed state, I began to see around the room. I looked down at my body and noticed I was ... detached. **Wow, can I go through the roof? Seemingly by my own intention, I did float above the roof.** I looked around, recognized the street layout, etc. This was at night, but I could make out the layout by the lighting. Saw the highways, leading to the interstate, saw the lights above the next town and just zoomed down I-20 to my home town in Louisiana. I saw my mother-in-law's house, where my wife was visiting, and lowered my self down through their roof into the living room. It seemed very bright, I saw her Dad at the dinner table, her brother watching TV. They apparently had just finished eating dinner and my wife was at the kitchen sink. I noticed that her Dad seemed to be looking directly at me, starring intently. I didn't know if he could see me, and thinking I didn't want to scare them, zoomed back up through their roof. While up in the sky, I looked at the moon - zoomed to and past the moon. The heavens and stars was beautiful, and I kept traveling. It seemed I was just a small ball of light, because sometimes travelling so fast I could see it move in front of me. Looking back to see where the Earth, and where I was, it was so small and a thought occurred to me. If I loose sight of the Earth, I am lost. So I kept my sight on the Earth, zoomed back fast, saw the USA, Texas, the highways, my house, and with a big breath and sigh of relief raised up off the floor and said ... What the Hell was That??? [sic] (OBERF)

After several weeks of trying to have another OBE, I awoke about 1am, which is quite common. I then try and reach a meditative state to have an OBE. A lot of time unsuccessfully. This time about 2.30am I felt myself falling into this state and I know that because my body started to vibrate. I felt totally at ease and relaxed and let the experience take over me. I then felt myself leaving my body, seeing myself on the bed as I exited out of my home. **This time was different as I felt totally in control. I went on several visits across the UK looking at different buildings and landscape and then onto a train station**. I could see the name of the station but couldn't quite make it out. It began with "S" and was 4/5 letters to long. Victorian looking building so would have been quite rural. I then went onto what I think was a farmyard when I encountered another person, a male, and usually people don't see that I am there however this person noticed that I was there and they were not happy. Is this possible that you can meet someone else during this experience? I don't really know. However they became threatening and started chasing me away following me for some time before retreating. I guess it was quite unnerving as I have not read about this being able to happen. I then travelled to another town and came across a female. We noticed each other and she approached me and gripped my shoulder. I could feel the touch as if it was actually happening. I assume that it was so I reached out and touched her hand to make sure and sure enough it was real. I didn't know what to think so quickly took off and went upon my journey exploring other locations. The whole experience seemed to last for about an hour or so at which point I felt exhausted and the I returned to my body watching myself slowly descend back into myself as I lay in my bed. (OBERF)

All experiences have been while I was lying in bed trying to go to sleep. The most recent is more of a wash than I would like. I was trying to pull "myself" out of "myself" but couldn't. I was fully aware that my body was separate from the part of me trying to "get up". I tried three or four times to get up, but gave up and decided to just sleep. I'm trying desperately to repeat my experiences and was actively trying to go "walking" out of body. The previous time was the most fun and I guess you could say educational for me. As with all other times, I felt "myself" shedding my physical body. **This experience is the time I learned I could control my actions. I walked out of my bedroom and went toward the rear of my house toward the kids rooms.** I walked into each of their rooms to check on them! **Afterwards, I knew I could have some fun and went outside...** only not through a door. I just went up. I did notice that everything in the attic was quite clear to me and was surprised at the detail in which I could see everything considering that there is no constant source of light up there. I realized in the experience prior to this that I could ( I know this sounds corny) FLY! Of all the things I might could have accomplished, I chose flight. Not the most responsible choice, but I wasn't thinking about responsibility at the time, only about the fact I could control myself. The strange part of this to me is how my experience ended. I read a lot tonight in the FAQ section of this site. The "silver cord" thing piqued my curiosity. I had no cord (none that I was aware of anyway) but was PULLED back to my body! I was above my house when I felt the pull and immediately came back down through the roof, through the attic, and into the hallway. I had no desire to end my little outing and was trying to keep from going back. I could feel my feet being pulled across the carpet in the hall and across the hardwood in the living room and dining room. I was pulled through walls though and only felt a kind of tingle as I passed through them. As I got back to my body, I realized my struggle was pointless and let it pull me back. as soon as I entered my body I was conscious again. I'm always awake and alert as soon as I "re-enter" myself. This was over a month ago. [sic] (OBERF)

# ^11^ The following story illustrates a situation in which a person exhibits both passive and active attitudes within the same LD:

**At the beginning of this dream, I am the conscious observer. I see myself sitting in a large banquet hall of a castle. I am sitting at a long wooden table with a group of men and women**. We are at a feast, and it seems as if a group of nobles or other royalty has come to visit and dine with us. I hear us discussing something about a wedding, an arranged wedding. I believe it is mine. I must be a princess. Then, the banquet ends, and I leave the hall with two of the men. It is at this point in the dream that I cease to be the conscious observer and become an active participant. We walk down a hallway to my left and then make a right turn. I notice the hallway is lit by a super bright white light with a hint of shimmery gold. As we walk, the light continues to grow in strength and brightness. I realize after awhile that we are walking downward as if the floor were on a gradual downward slant. Finally, we reach the center of the castle deep underground. I see a man standing guard in front of an open pit. I see flames coming up from the pit. I need to retrieve something from the fire pit. One of my men tells the guard to step aside, and he does without argument. My man goes and stands next to the guard. They talk quietly while step forward toward the pit. My second man is right behind me. I walk in thin air above the pit and flames. The heat from the flames does not bother me at all. **I then descend as if by magic down into the pit. I am completely engulfed by fire, but it doesn't bother me. My right hand moves forward and grasps a large object**. The object is oval-shaped and about twice the size of my hand. It is green and hard like a stone. At first, I think it is a stone. Maybe an expensive peace of jade or something. Then, as if by magic, I slowly ascend out from the flaming pit. I float back to the pit's edge where my man waits for me. We step away from the pit and walk back to the guard and my other escort. The guard goes back to his station by the pit, and the three of us begin our long walk back up to the castle. As we begin our journey, I notice the green stone in my hand is glowing with a soft emerald green light. It is still warm, perhaps from the flames, but the warmth makes me think it is alive. I can feel a slow and steady pulsation from its center, like a heart beat. I realize I am not holding a precious piece of jade; I am holding a dragon egg. Furthermore, I know immediately that the dragon egg is ready to hatch. [sic] (LDE)

# ^12^ The following excerpts exemplify reports of realism commonly found in accounts of OBEs:

I had been out of town for a funeral. My aunt had just died unexpectedly, and I was close to her. She had helped raise me early on in life, and was a very vocal and strong family advocate. I had been home two days when the OBE happened, and I still question whether or not it happened. This is the first time I have had an experience like this. I really don't sleep through the night because of incontinence, and got up to go to the bathroom around 2 A.M. As I got out of bed, and started walking to the bathroom, I noticed something in my bed. I stopped and looked, and my body was still lying asleep in bed, next to my wife. I was awake, and knew I wasn't asleep, but I could still see my body lying in bed. I stood frozen next to myself, not knowing what to do. I called for my wife, and she responded, telling me to leave her alone she was sleeping. I asked her to shake me to rouse me and wake me up. She said, obviously you're awake if we're talking. I stood there looking at myself, knowing I wasn't dreaming, and the fact that I had just gotten a response from my wife reassured me that I was awake. I have sleep apnea, and often mess with my mask while asleep according to my wife. While I stood there, I watched myself adjust my mask and turn over to reposition myself. I could hear my teeth grinding, and watched myself put my arm over my wife's side to hold her. **This whole scene was very real**, and I know I wasn't sleeping. I wasn't worried or scared at the time, I was actually very comfortable with what was going on. I stood there and watched myself sleep for some time, then finally decided to touch myself to see if I would wake up. When I reached to put my hand on my shoulder, I woke up before I actually touched myself. When the sleeping me woke up, I was startled. My wife felt me move, and already stirring because I had called for her, she said would you just go to sleep and leave me alone. At that point, I realized I was within my body, and no longer watching. The next morning, my wife expressed frustration that I had called to her and woke her up, and said that I was loud and seemed irritated myself, almost worried. I told her what happened and she said I was dreaming, **and it just felt real**. [sic] (OBERF)

# ^13^ The following excerpts exemplify reports of realism also commonly found in accounts of LDs:

[…] The dream was very clear in my mind; I was thoroughly awake; I perceived its great interest to me, and I stamped it on my mind — I venture to say — almost exactly as I tell it here. (Green 1968)

I dreamed that my wife and I awoke, got up, and dressed. On pulling up the blind, we made the amazing discovery that the row of houses opposite had vanished and in their place were bare fields. I said to my wife, ‘**This means I am dreaming, though everything seems so real and I feel perfectly awake**. Those houses could not disappear in the night, and look at all that grass! But though my wife was greatly puzzled, I could not convince her it was a dream. ‘Well,’ I continued, I am prepared to stand by my reason and put it to the test. I will jump out of the window, and I shall take no harm / Ruthlessly ignoring her pleading and objecting, I opened the window and climbed out on to the sill. I then jumped, and floated gently down into the street. When my feet touched the pavement, I awoke. My wife had no memory of dreaming. As a matter of fact, I was very nervous about jumping; for the atmosphere inside our bedroom seemed so absolutely real that it nearly made me accept the manifest absurdity of things outside (Green 1968).

It was a night of disrupted sleep; I woke up at 5am to the persistent sounds of a mouse and couldn’t go back to sleep until 6:30am. I was so exhausted that even before falling asleep again, I could see flashes of hypnagogic imagery or “mini dreams” in my mind’s eye. I gently observed them pass by, until one popped up that was especially clear... a slice of raisin toast! At first the image was slightly fuzzy, as if floating in a bubble. I focused on it and imagined zooming towards it. I asked myself: What texture does it have? Is it crispy or soft? How does it smell? I examined each granular detail. As I did so, the image became sharper until suddenly it was under my nose. When I felt I was fully in the dream, I shifted my focus to my reality check bracelet... the usual letters had vanished! It worked! I’m dreaming! I found myself sitting up in bed in a basement, with a loaded breakfast tray beside me. **It felt so realistic, right down to the feel of the sheets against my skin. It truly seemed I had woken up in a parallel dimension, vivid enough that it raised the question of which reality was real; who was dreaming of waking up and who was actually awake?** It was a fascinating feeling; the transition from wake to dream was so smooth, there was no clear sign as to when it happened. It felt as subtle as if I had just leaned forward slightly, except that it was my awareness that had moved instead of my physical body. After stabilizing the dream by spinning around, I explored assorted objects in the room and then the nearby neighborhood, delighting in the lucid wonder of it all. I woke up briefly and went back to sleep. The same technique worked again! As I relaxed once more, the mini dreams began appearing before me. I saw my grandparents’ living room, as it was in my childhood. I could see the Christmas tree at a distance and imagined zooming toward it. I felt like I was partially there, but needed more details to mentally latch onto to pull myself fully in. However, the tree was blank... So I visualized more details into existence by asking myself questions like: Don’t you see an ornament just under that branch? This pulled my curiosity further in and pushed me to look just a bit further to see more. In doing so, my unconscious created more for me to see. As I visualized my grandmother’s ornaments one by one, they slowly appeared on the tree. As they materialized, I looked closer at each one. If there was a detail missing I would think: This one had more of a jagged texture; doesn’t it feel like that? and rub my finger along it, fully expecting to feel that texture. I have such clear memories of each ornament from my youth that it was easy to recall their details. I maintained focus like this until the dream finished forming around me; once more, the shift from waking visualization to being in the dream scene was seamless. I stabilized my lucidity even further by spinning around and rubbing my hands together, and then went on to have a heartwarming reunion with my deceased grandfather! (LDE)

# ^14^ Examples of LDs involving realistic representations of individuals and familiar environments:

If I saw in my dream one of my friends whom perhaps, I had not seen for several years, **he spoke to me in his own language, in his own voice, with his own intonations and inflections, with his own characteristic gestures; and he said precisely what only he could say**. Every man has his own manner of expressing himself, his own manner of thinking, his own manner of reacting to outward phenomena. No man can speak or act for another. And what first attracted my attention in these dreams was their wonderful artistic exactitude. The style of each man was kept throughout to the smallest detail. It happened that certain features were exaggerated or expressed symbolically. But there was never anything incorrect, anything inconsistent with the type. In dreams of such a kind it happened that I saw more than once ten or twenty people simultaneously whom I had known at different periods of my life, and in not one of them was there ever the slightest mistake or the slightest inexactitude. (Green 1968)

One of my first lucid dreams in my early 20s was of a healing nature. In the **dream I was sitting in a room with my parents and their acquaintances, and we were all sitting and watching TV. At the same time I noticed that my mother was talking over the TV (as she often did in real life) which at first I thought was annoying**. Tuning into what she was saying made me really embarrassed as I realised she was saying judgemental things about me. Things I didn't want the other people to hear. I felt betrayed, humiliated to my very core, and angrily stood up and left the room. By the time I walked into the next room which was dark, I was already crying from helplessness. The next thing I noticed was the same TV set as in the previous room, which struck me as odd and I realised I was dreaming. As I sat there in the dark room next to the TV set, aware I was dreaming, I couldn't feel excited about being lucid because I could still feel the emotional pain that forced me to run away. Instead I had an idea to find exactly where the pain I felt was located. Still sobbing I took a deep breath and tuned in to my body. I felt the pain in a knot in my chest and heart. I felt determined to work with this pain as much as I could in this dream, to release it. Simply allowing it and feeling it was the first step. It wasn't pleasant and it brought more tears but it felt like the right thing to do. Then I wished to breathe it out and it happened very swiftly. As I started to relax feeling relieved, I woke up on a pillow wet with tears. The relationship with my mother was never the same again after this dream. Later the same morning I noticed something had shifted in me and in her. I wasn't so attached to her moods, opinions and judgements. I felt compassion for her. This was a powerful beginning of healing my mother wound. [sic] (LDE)

# ^15^ An example of an OBE story depicting an environment congruent with the physical world:

As a very small child I heard about hell, and worried, though I didn't talk yet. May have been three I think. I remember I went out behind the woodshed and there was a wheelbarrow. I thought. "If I can push this wheelbarrow over, I will be saved." I knew it was just a made up thing, I had no such information given to me, just trying to somehow settle this horrible thing of not wanting to go to this forever fire of hell. I tried and shoved and after while I accomplished to push over this big wooden wheelbarrow. I did not rejoice at being saved then, I knew it was just made up thing. Whether this time or another time, I was at the same place out behind the woodshed. as I walked, I began to see strips of pink and yellow light on the ground. I don't think it was moving light, just strips of pink and yellow light on the ground or the air tinted pink and yellow on the ground as I saw it. The next thing I remember, I am way up in the sky. It is as if I am in an airplane looking down at the earth. though as far as I know I was just up in the sky looking down. **I saw the roofs and buildings, and the ground and myself on the ground very small, like an inch or so high, but I could see myself exactly as I was, and the same as I was dressed, still like walking behind the woodshed**. Only a one glimpse,/still life thing. like a picture I saw. Then all of a sudden I was again in my body. [sic] (OBERF)

I fell asleep telling myself I would try to induce an out of body experience (OBE). I became consciously aware of my self while in a dream and sat up in bed. I stood up next to my bed and began to realize that I was separated from my body. **My room was exactly as it is in reality**. **It was dark, the glow of a street light coming in from the window was there. It was very real.** I turned to see myself lying in bed. I saw the blanket and the bed and my general form and then became very frightened and did not allow myself to look at my face. I tried to scream for my wife. I was paralyzed, unable to scream and closed myself out of the whole experience. I woke up after a few seconds of absolute terror calling my wife's name (she was in bed next to me). I spent the next few days scared to go to sleep like a child afraid of the dark. I knew I needed to overcome this, but the fear is very real. [sic] (OBERF)

# ^16^ Examples of OBE stories depicting an environment incongruent with the physical world or that is mutable:

I had stayed home from work that day because I was congested and feeling under the weather, but was still taking the prescribed pain killers for my back as I had everyday for sometime by this point . Towards the end of the night I had a glass of jack and coke which my live in boyfriend at the time made a comment about that being foolish to do when I’m sick. So after that drink and watching television with him for a while, I told him I’d go to the doctor in the morning to see what was wrong with me. Then I went into our bedroom alone to sleep. […]. After I thought I was asleep I for some reason decided to get back up. I walked through the bedroom door to my boyfriend who was watching football with lamp on next to him. I Walked to him saying, 'Babe I can’t sleep.' He ignored me, didn’t even acknowledge me so I got aggravated said his name again. Then said it again this time seething in frustration saying it loudly. Then I felt a presence move behind me so I looked and at that moment saw my body in the bed. I was overcome with fear. I quickly scanned the apartment taking note it was as it always was, but **saw the window to the outside of the apartment didn’t have the usual apartments across the hall Instead it was a purple darkness**. I then thought in my head this must be hell. I turned to my body with complete fear and ran to my body. I then was in my body jumped out of bed and told my boyfriend that I believed I just died. He scoffed so I never spoke of it again. **I noticed something odd which was that the closet door to the bedroom was open when I came back to my body, but was closed prior to my OBE**. Also the next day I was diagnosed by my doctor with walking pneumonia which isn’t a big deal, but with the pain killers and the drinking I always felt perhaps it was a near death, but at this point I’m not sure and all I am positive of is that life exists outside of this physical body. I also had a couple other experiences as a child and even in the womb. I remember these experiences like they happened yesterday and my memory isn’t great so that’s how real and profound they are. [sic] (OBERF)

I laid down to take a nap one afternoon and as I was drifting off, I heard a loud buzzing all around me. This had happened previously and I always shook my head to get rid of the sound. This time, however, I decided to just let it go on. The next sensation was of falling. I kept telling myself that I was safe and to just let it happen. As I was "falling," I heard voices that I didn't recognize. It sounded like bits and pieces of conversations. Then suddenly I was floating up toward the ceiling. I remember thinking that this was impossible, that I wasn't light enough to float. I ended up at the ceiling, kind of bobbing around and I felt really free. There was no fear at all. It felt totally natural. But when I looked down, I saw a little blond girl in the doorway. She was looking up at me and pointing. Then a woman came into the room, looked up and grabbed the little girl's hand and they both left the room. I didn't recognize either one of them. **I felt like I was in my bedroom but it was different. For example, my pictures were no longer on the walls. I felt like it belonged to these other people that I have never seen before.** I almost felt like an intruder. Anyway, after they left I found myself back in my body on the bed. I was totally awake and this experience was very real and vivid. This experience was NOTHING like a dream. [sic] (OBERF)

Also I noticed that though I might fly around the neighborhood, **I unintentionally "changed" things**. For example, if I decided to fly through a house, I might find a window to fly through where no window exists in waking reality. Upon waking and recalling the situation, I would note that I had unknowingly made it easier for myself to fly into the house by mentally perceiving a window where none existed. (Waggoner 2008)

I remember it was early afternoon. I laid down for a nap because I was tired. This was unusual for me, as I rarely took naps by my own choice, but I felt very tired and figured I would lay down for a bit. Just as I was dozing off, before I entered any dream state, I felt myself floating out of my body. I looked down, and saw myself lying on the bed. […]. As I floated towards my bedroom, I passed my mother's room, and I felt a strange, convincing force drawing me to go inside of that room for some reason. I opened the door, and looked inside. **It looked normal, but felt devoid of any human presence or energy**, as though it was cut off from the human realm. I remember entering the room, and thinking it was just my mom's room, so what was the big deal about me going in there. I turned around (facing the door) to close the door (I felt a strong urge that I needed to close the door and remain in that room). **I was facing the door when I closed it, and after I did, I turned around, and it was no longer my mother's room, but a long, narrow room, medium blue walls ceiling and floor, and completely empty, except for 2 strange figures at the end.** As I moved towards them, the room kept expanding. It seemed like hours until I reached the end. When I finally reached the end, the 2 figures were waiting for me. They had blurred faces, communicated telepathically, and were wearing what looked like civil war colonial dress uniforms. One was stout, sitting at a desk with his head down, writing in some large, mysterious book with a feather pen. […]. **To my shock, when I turned around, I was directly in front of the door, and what I saw of the room looked normal. I opened the door, looked back, and what I saw was those 2 figures, far away, and the room looked like a combination of the blue room, and my mom's room as I knew it, as though 2 dimensions were melded together**. It's very hard to describe this scene, but I really have no other way to describe it. I remember leaving that room, floating to my room, and re-entering my body. I really don't know if I went back to sleep at that point, but when I awoke, I knew this had been more than just a dream, and that experience stuck with me for the remainder of my life. [sic] (OBERF)

# ^17^ OBE stories showing dream-like features and/or encountering with “unreal” characters:

I moved towards the window. My surroundings seemed dim. **The walls appeared to approach me, then disappeared**. Nearby I saw a friend. He spoke, or perhaps he did not use language, though I understood: "Did I see where I was?" Yes, I could see though the sunshine had faded and we were in a narrow road. **Gloomy overhanging rocks were on each side, obstructed here and there by projections which seemed to block up the passage**. It was dark and cheerless, surrounded by cold mist. ... T looked farther. Afar off, a brilliant gleam of light burst out, flooding the road with unconceivable glory. ... We turned aside. ...A sense of motion, increasing light, intense living radiance, and then - Who can describe the indescribable? Time had disappeared, space no longer existed. I knew that I was a part of this undying, infinite, indestructible whole; that without me it would not be complete. The light of this great life penetrated me, and I understood that thoughts were the only real, tangible substances, and why, between my friend and me, utterance was not needed. The secrets of life and death were unveiled. The reasons of sin and suffering were evident... **I saw living, radiant beings** ... while there were others for whom I felt an intense compassion and an irresistible desire to draw them nearer to me. […]. Something in the vaporous mistiness, in the forms and shapes, recalled the dream-life: **I knew that in this region the dream-life was lived. ... But I wondered why the objects were so different: these rocks, which before had seemed so solid were only vapours or clouds through which I now passed without resistance** [sic] (Crookall 1966)

The vibrations begin. I’m floating out of my body and about to exit through the window. I end up going up to the ceiling. I wonder if I’ll be able to make it through this time. I get stuck. I remember that willpower is powerful in this state, so i tell myself I can push through. Sure enough, I break through. I’m outside and I immediately feel the cool, humid air. It’s night time and it just recently rained. I’m in an alley in between brick buildings. **I say aloud, “there will be a portal to my past life around the corner”. When I turn the corner, I see a glass tank about the size of a fish tank**. **There is a glowing white mist with sparkly particles in it. This must be the portal.** I look into it. **A fish forms. It’s swimming. It turns into another fish, and then another, and then another that looks like dory. Then it slowly turns into other land creatures. It’s like one creature quickly evolving through aeons of time**. [sic] (DreamJournal)

[…] I paused, trying to think of a good experiment. "I know," I thought to myself, "I'll try looking into a mirror!" As soon as I thought that, I felt a strange shift of consciousness and I opened my eyes. I found myself in a strange room with a mirror. I walked over to the mirror and looked at it. At first I saw my own image, but without a beard-I only had a moustache. […] **So I smiled into the mirror and watched my image change. My image changed slowly into the image of a woman I've never seen before**. […] (Peterson and Tart 2013)

[…] While in the process of washing my face with cool water I began to hear what sound like a very soft, sweet harmonic choir all voices no music. It sound as if there was a million of them each voice or group of voices all uniquely distinctive OOHs and AH. were the only words to the song. The most beautiful harmonic sound I have ever heard in my life. I often keep my radio in the bathroom on soft classical music so the first thing I did was check to see if the radio was on. Of course it was not on, I could still hear the voice so they must have been coming form the radio in my bedroom; I then walked out the bathroom to check the radio on my nightstand near my bed and that's when it happened to me. It was like my spirit was called from my body I could feel it leave as if it just floated out of me like a helium balloon. While my body was frozen in the room in a standing position I did not look back at myself the frozen stiffness was a feeling. This floating feeling was like a swift journey, I quickly appeared in a place where only a few things were visible and familiar. I heard the voice the whole time; which as you recall this experience began with what I like to refer to as a heavenly choir singing. **I recognized two enormously huge dragons on each side of two very large gates of what looked like ivory or pearls with gold brackets**. Although at this time I don't recall the exact location of the gold on the gates. The gates were open but I was not allowed to enter. A voice was talking to me telepathically and I remember calling it LORD. **There were clouds light and the heavenly choir.** During this telepathic conversation; which seem to be filled with an enormous amount of information, non I could remember except one, only one reply and one answer stuck in my memory bank like learning to ride a bicycle. **I remember trying to enter the gates to get a closer look at what was in side by the dragon on the left with the statue appearance moved in a very subtle way.** I felt it was a signal to say I could not enter. I looked down to see I was standing on a cloud. Clouds were everywhere, I said LORD how can I stand on a cloud, I can't stand on a cloud! The reply I received was swift, sharp, soul piercing, and quickly returned me to my body. I did not have time to look at myself but I could feel my soul return as the wind make the leaves move on a tree. I could move again, my sight was back in my bed room I could still hear the heavenly choir. [sic] (OBERF)

I woke up in my dream leaving my body quickly. This time everything was different. I wasn't scared anymore. I was somehow prepared. I had made a decision about where to go if it happens again. I went to some friends of mine. They were sleeping at the time. They are a couple. The man usually works night shifts, he was there, and in the morning I checked them and he really was there. However, I was in their sleeping room. I tried to throw a frame of a picture but couldn't. I only felt the cold glass. I went out. There was a fog and I couldn't see much. Then I told myself I wanted to look around. All of a sudden it all became clear. It was a sunny day. There were a lot of people, talking, shopping. **The place was the same without blocks, but with houses. But the streets were the same. There was a tree, which was very strange, I have never seen something like it before - an ordinary tree, but with different leaves - very though, they resemble the leaves of the bushes that are cut in a square form**. I floated above the people and then I found myself on a different place. It was gloomy, there was a lot of mud, there people but they looked strange. they were working. **All of them were wearing brown clothes. I saw a woman with a head of a fish.** This was the strangest of all. I then went back to my body. [sic] (OBERF)

# ^18^ Robert Monroe’s stories “traveling” to nonphysical realities and encountering human-like beings:

[In the OBE world] the scientific development is inconsistent. There are no electrical devices whatsoever. Electricity, electromagnetics, and anything so related are non-existent. No electric lights, telephones, radios, television, or electric power. No internal combustion, gasoline, or oil were found as power sources. Yet mechanical power is used. Careful examination of one of the locomotives that pulled a string of old-fashioned-looking passenger cars showed it to be driven by a steam engine. The cars appeared to be made of wood, the locomotive of metal, but of a different shape than our now obsolete types. […] Traffic is not heavy. Self-powered vehicles exist in the form of a four-wheeled platform which is steered by the feet acting upon the front wheels. A mechanism pumped by the arms transfers the energy to the rear wheels, much like the children's "rowing wagons" of some years back. (Monroe 2014)

[…] I was just about eight inches over the physical when I saw a movement out of the corner of my eye. Walking up beside my body was a human-appearing body (I could see only the lower half with my head in the position on its side, by turning my eyes to the right). It was nude, no clothes, and male. He seemed in size to be about ten years old, some three feet tall, thin legs, little pubic hair, undeveloped genitals. Calmly, as if it were a daily occurrence-like a boy swinging onto his favorite horse-he swung a leg over my back and climbed on me. I could feel his legs around my waist, his small body pressed against my back. I was so completely surprised that it didn't occur to me to be afraid (perhaps his size had something to do with that)! I waited rigidly, and by rolling my eyes to the right, I could see his right leg hanging over my body, less than two feet away. It looked like a perfectly normal ten-year-old boy's leg. I was still hovering just out of the physical, and cautiously wondered who and what this was. […] It was certainly humanoid in form, but upon reflection, it didn't have the feeling of human intelligence. It (he) seemed more animal, or somewhere in between. (Monroe 2014)

# ^19^ Examples of LD showing environmental stability or congruency with the physical world:

I fell asleep. **I could see clearly all the little objects which decorate my study. My attention alighted on a porcelain tray, in which I keep my pencils and pens, and which has some very unusual decoration on it**. ... I suddenly thought: whenever I have seen this tray in waking life, it has always been in one piece. What if I were to break it in my dream? How would my imagination represent the broken tray? I immediately broke it in pieces. I picked up the pieces and examined them closely. I observed the sharp edges of the lines of breakage, and the jagged cracks which split the decorative figures in several places. I had seldom had such a vivid dream. (LaBerge 1986)

**In previous lucid dreams, several times I arrived at the balcony of my childhood home, an apartment at the 5th floor**, with the desire to jump and fly, but I never reached the courage to take the leap. Even after double-checking that I was actually dreaming, throwing myself off that balcony didn’t seem a very good idea. Then, on December 2nd 2019, in my present home bed in Madrid, I have the following experience: After a failed WILD attempt – the vibrations wake me up – I manage to stand up within a dream, fully conscious. I am in a room that is not mine. I move out into a dark hallway with beautiful colors in the background and, without having it planned beforehand, I ask aloud to ‘the awareness behind the dream’ “How can I work the limits?” On the hallway wall I see some letters written in light, something like ‘galata’ in Greek, but they change and I cannot read them well. It seems that the dream is getting blurred, but I move fast and I recover it. While I keep asking the question, I reach the shuttered door of a balcony, I raise the shutter – **the noise is identical to the one of my childhood home!** – and I just jump off the balcony without any thinking. Enjoying the fall, I lose consciousness and wake up.. [sic] (DreamJournal)

On 1pm, I was preparing for a short nap, but was woken up after almost falling asleep. I moved my sleeping spot and went to a noisier and more uncomfortable spot because I was asked to. I went back to sleeping shortly after that. I then dreamt of my school cafeteria with a few of my classmates there. I walked around a shelf pillar multiple, mainly to distract myself because I was too nervous to talk to my classmates. I later found out that this was a dream, so I assessed the room properly and thought, "Wow. Theres so much details in on this shelf pillar". I saw that the shelf was full of items. The only items that I remember were a tiny camera and a metal watch. I was then curious what was inside of my classmate's backpack. I ran towards their backpack and unzipped it. **Upon seeing the inside of their backpack, I was shocked... It looked exactly like the inside of my backpack**. **I then searched around the room, looking for another bag, and saw a shoulder bag. Immediately, I ran towards it and opened it. I saw that the inside of the bag looked like the inside of my mom's purse**. (After that, I had a different dream, but i wasn't lucid dreaming anymore) [sic] (LDE)

# ^20^ Some anecdotal examples of alleged information acquisition during OBEs include cases where individuals have reported observing objects or events in distant locations that they could not have known about through their physical senses:

The mall was packed during the holiday season. Hundreds of people raced in and out of stores trying to complete their Christmas shopping. Upon discovering that my 3-year-old daughter was missing, I started getting a sick feeling in my stomach. I turned and walked out of the store. As I walked into the mall area and saw the rush of people going by, I went into a full-fledged panic. Seeing a toy store across the hall I ran to it hoping my daughter wandered there to explore the toys. Just as I made it to the doors, the clerk was locking up. Instantly, the most powerful fear erupted inside my body as I thought of the stores closing and people beginning to leave the mall. The most horrible stories about people who had lost a child began to play like a movie in my mind. I could not stand it, the thought of my child with someone I did not know. I thought I could hear her crying, and feel her fear. Something strong and overpowering was taking over my body and mind. I kept thinking, "No, this can't be happening to me. I don't want to be one of those people who wait for any news about their child." I could feel the sickness in my stomach. My legs were tingling, and a strange feeling was moving through my body. Suddenly, as if my spirit had left my body, I could see myself standing there alone. "Am I dying? Will this be forever?" Everything seemed bright, like everything and everyone was associated with a light of it's own. The noise, a rumbling noise, as if people's voices were melting together into one echoed in my head. And, a vibration, like the mall itself was feeling the pressure from my energy. **All of the sudden I was directed by my spirit toward the far end of the mall. I turned and searched with all my power spotting my daughter**. My legs went numb, my breathing was heavy, and my vision was blurred from the tears. I thought I was going to pass out just from the emotional change. It was the longest time of my life. [sic] (OBERF)

I was asleep and I could hear a loud, very loud mechanical machine. It sounded like a roller coaster ride starting up. I left my body and floated down the road. I could see everything as it was but from above. I knew I could go anywhere in the world and see anyone that I wanted to see. I knew I could go as far as I wanted to go, but I didn't want to go far away because my kids were sleeping beside me. I don't know why it happened, but I realized that there's plenty of freedom when you leave your body. On a different occasion about 4 years ago, I was asleep and had a similar experience. My son was 16 years old and moved in with his dad a few states away. I missed him terribly and one night I had the same thing happen where I could leave my body. I went to my son and saw him sleeping. I told my self I can't be with you, but at least I can watch you while you sleep. **A few months later I saw my son and told him about my dreamed and described his apartment that I had never seen before in detail, including the size of his bed, what his room looked like and even the color of his sheets. He said I was exactly correct**. [sic] (OBERF)

# ^21^ Examples of shared OBEs:

[…] With regard to other experiences, Mrs. Joy further said, **'I was seen by one of my neighbors when I was ‘out’: she saw me enter her cottage** - I have done this many times and she thought it was a ‘vision’ of me. ... I also visited my aunt in this way on several occasions. I touched her once and she looked round, worried. **She knew who it was but could not see me**.” (case 97) (Crookall 1966)

I entered a bedroom where my friend was sleeping, approached her, and, intending to awaken her, pulled her arm. Then I remembered that I must be dead, and it would startle her if she saw me. So, I started to turn away from the bed. **Suddenly she opened her eyes and saw me**. When my friend returned from the funeral, **she told how she had felt me pull at her arm, awakened, recognized me, and then saw me vanish**. (case 100) (Crookall 1966)

# ^22^ Anomalous cognitions can also occur in the context of LDs:

I became aware that I was dreaming and decided to try to communicate with my son. I had an impression that contact had been achieved and attempted to convey to him the words, ‘I can’t stay long; I am feeling muzzy.’ **When I met my son the next day for lunch he repeated these words to me before I had mentioned the matter to him and said that he had received the impression in a dream** in which he also was aware that he was dreaming. (Green 1968)

In May, 1905, I dreamed that I was in a little provincial Dutch town and at once **encountered my brother-in-law, who had died some time before**. I was absolutely sure that it was he, and I knew that he was dead. . . . **He told me that a financial catastrophe was impending for me**. Somebody was going to rob me of a sum of 10,000 guilders. I said that I understood him, though after waking up I was utterly puzzled by it and could make nothing of ,it. . . .I wish to point out that this was **the only prediction I ever received in a lucid dream in such an impressive way**. **And it came only too true**, with this difference, that the sum I lost was twenty times greater. At the time of the dream there seemed not to be the slightest probability of such a catastrophe. I was not even in possession of the money I lost afterwards. Yet it was just the time when the first events took place — the railway strikes of 1903 — that led up to my financial ruin. (Van Eeden 1913, Green 1968)

I was walking in a country lane at A., the place where my parents then resided. I was reading geometry as I walked along, a subject little likely to produce fancies or morbid phenomena of any kind, when, in a moment, **I saw a bedroom known as the White Room in my home, and upon the floor lay my mother, to all appearance dead**. The vision must have remained some minutes, during which time my real surroundings appeared to pale and die out; but as the vision faded, actual surroundings came back, at first dimly, and then clearly. I could not doubt that what I had seen was real, so, instead of going home, I went at once to the house of our medical man and found him at home. He at once set out with me for my home, on the way putting questions I could not answer, as my mother was to all appearance well when I left home. **I led the doctor straight to the White Room, where we found my mother actually lying as in my vision. This was true even to minute details.** She had been seized suddenly by an attack at the heart, and would soon have breathed her last but for the doctor’s timely advent. (Green 1968)

^23^ Example of shared dreams:

In the spring of 1978, I was attending a small Midwestern college. I was friends with a group of five young women who occupied a dormitory suite at the other end of our co-ed dorm. We were friends only, and I never felt any romantic interest in any of them. **One night I dreamt that I was in their suite, and I went from room to room and made love to each one.** It wasn't passionate/lustful lovemaking; it was more like sharing a soulful experience, and communicating with each other our deepest thoughts and emotions. In fact, I clearly remember one of the women of the dream telling me how extremely unlovable she felt, while I reassured her. Later, I learned that this woman had a very unhappy home life. The next day I remembered the dream because it was so vivid. As I was walking to class that afternoon, someone in the dorm came up to me and said, "Hey, I heard you were quite a Romeo last night." **I asked her what she meant by that. Then she told me that Nadine and Sheila both dreamt that I made love to them in their dorm rooms the night before. I was amazed! Three of us apparently recalled the same dream incident!**  I was too shy to talk to them about it, and I was also concerned that they would interpret the dream on a physical level, instead of the deep emotional level that it was to me. Now I wish I had talked to them and gotten the details of their dream experience. (Magallón 1997)

# ^24^ There are accounts of OBEs that involve sense of non-existent time:

I want to share a experience with you that was the most beautiful, joyous, wonderful day of my life, […]. So I'm now on my way into clearing my mind, when a sudden rush of energy comes over me and it feels as if my hair is standing on end as well, I remember I had my hands clasped where I could feel my fingers really well, this was different it where as if it where someone else's hands and fingers, they felt so weird just as the experiences […] The color was so vivid nothing like I had ever seen here before. It was of orangey hues so vivid so admirable. […]. I remember seeing a light coming toward me, it was bright but not like a white bright more of a white with a mixture of yellow and orange mixed amongst it. Immediately, I knew this light was my Dad! We walked hand in hand along the beach talking and catching up on things, although, I can't remember what all was said. At this point, it was more like two lights band together holding hands. **We talked like seemed like forever but yet there was no awareness of time here. It is hard to picture this, as everything here on earth is run by time itself. There's just no feeling of time there.** I truly can't even explain it myself. After taking our walk together he and I flew around in the sky playing and dancing as if we where two little kids having the time of their lives! At this point, he was my father. But then again, he wasn't; he was just a child to me when we were playing. We had a blast! I can't remember ever having this much fun and feeling this free and full of so much love and peace! When it was time to go, he gently lowered me back down through what seemed to be clouds.[…][sic] (OBERF)

I laid on my stomach peacefully, arms under my pillow and my head resting against the pillow. I was waiting for to fall asleep and I felt myself very relaxed. Just like before you actually fall asleep. Suddenly I felt like someone would have took a hold from my ankles with both hands and started yanking my legs upwards, like trying to flip me over. I know my legs were on the mattress, so I though 'This feels rather strange, like someone's yanking me'. The force - whatever it was - yanked my legs pretty high, maybe 50cm/19,5 inches upwards, and on the third yank I flew out of my body. It happened so fast, it didn't even took a second! It felt like it all happened in 000.1 second. I think I came out through my legs, as I literally felt yanked out by the force which was playing with my ankles. **I found myself standing in a COMPLETE emptiness. There was NOTHING in there. It was pitch black, so black and empty that even the color black didn't exist there. There was no direction, no time, so sound, no smell, nothing. Absolutely nothing. There are no words in this Earth in ANY language which could describe this complete emptiness**. Even the word complete emptiness makes no sense, as emptiness is something that exists, but in that black space nothing existed. Nothing. It was overwhelmingly dark. I also felt that it was dense and limitless, reaching for eternity. I felt nothing on me or against my skin, but I just knew the place was thick somehow. I looked around in panic thinking 'Oh my god, I'm out of my body and I have no idea how to get back!!' […] I spend years thinking this scene might have been just my imagination and it never happened, as it sounded crazy - how can one see herself standing in the air from other person's perspective, like you sometimes see dreams of yourself where you inspect your dream self as an invisible watcher. [sic] (OBERF)

Sellers (2017) describes an OBE with altered sense of time:

According to Mr. M, prior to the onset of one of his regular daily [experiences] back in 2013, he looked at the watch at 2 P.M., just before leaving his body. Upon returning to his ordinary state of consciousness, he again checked the time, only to find that it was six hours later. Mr. M says, “**The 6 hours felt like 20 minutes to me. I really was under the impression that the time difference between the onset of the [experience] and the time I returned to the regular state of consciousness was about 20 minutes, not 6 hours**. At least this is how it felt to me. I was horrified.” (Sellers 2017)

# ^25^ The following examples demonstrate an altered sense of time in LDs:

On Sept. 9, 1904, I dreamt that I stood at a table before a window. On the table were different objects. I was perfectly well aware that I was dreaming and I considered what sorts of experiments I could make. I began by trying to break glass, by beating it with a stone. I put a small tablet of glass on two stones and struck it with another stone. Yet it would not break. Then I took a fine claret-glass from the table and struck it with my fist, with all my might, at the same time reflecting how dangerous it would be to do this in waking life; yet the glass remained whole. But lo; when I looked at it again after some time, it was broken. It broke all right, but a little too late, like an actor who misses his cue. This gave me a very curious impression of being in a fake-world, cleverly imitated, but with small failures. […] I then decided that I would try to reach a certain ruined temple in Tibet, of which my Master, Azelda, had spoken. With this end, I concentrated all my will in one big effort, expecting to rush off in some horizontal direction. **The result was absolutely unexpected. The ground collapsed beneath my feet and I was falling, with seemingly tremendous velocity, down a dark, narrow tunnel or shaft. This downward descent continued until I lost my time-sense and it seemed that I might have been falling for hours**. Something in me was geting frightened, but I managed to keep calm by telling myself that I was really in bed at Wimbledon and that my Master would protect me. At last I came gently to rest. Blackness and silence; then, as one awakening from a heavy sleep, I became progressively aware of my surroundings. [sic] (Green 1968)

During a lucid dream, I had decided to attempt an out-of-the-body experience, so I had partially fasted all day. I lay down on the bed and concentrated. After a time I felt myself falling, as usual at the start of these experiences. One seems to fall and then checks oneself and returns. After a short time I succeeded in letting myself fall but on this occasion I went on falling for much longer than is usual. I seemed to be going down through miles and miles of cotton-wool, and I had the feeling that it was closing me in and that I should never be able to get back. **Also, I had the idea that I was going back in the past**. Finally the fall came to an end and I found myself standing in the corridor of a monastery. It was not anywhere I knew, **but I had this feeling it was some time in the past.** [sic] (Green 1968)

# ^26^ The following examples demonstrate the emotional quality and positive after-effects associated with OBEs:

A month later it happened again. My heart beat very fast and again and it was like being absorbed by something. I was out of my body but this time I wasn't so afraid and I could thought with normally. I saw myself in my bed and I felt so free!! I went around of my bedroom and saw the window. I returned to my body. When again I could to move I felt so happy. It was like having experienced something unique. The life in Earth was nothing  compared to what have just happened. I was eager to return to live that experience. When it was happened again just time I was over a village over 6 kms away from there. I can not explain what I felt. I returned to my home by the beach, everything had a special glow. The waves were a special white color and the stars came out sparks of light like colours and gases. **A feeling of liberation and unity with the universe. I had many experiences like this, and my life has changed completely**. [sic] (OBERF)

I suddenly became ill in the stomach area and attempted to reach the ladies room. I did not reach it but lowered myself to the carpet in the study area because somehow I know that I would pass out and fall down if I didn't hurry up and get on the ground. It became black as night. **Suddenly tremendous peace filled my being and I was so content and happy and satisfied. Still didn't see light nor a tunnel but just so peaceful. I felt great love, just like someone loved me** all over NOTHING like earthly lust or even love, I did not see anything but blackness but I loved it! And something in the distance let me know I had to go back and wham I jolted back in my body to find a sweet oriental young lady over me asking if I was ok. [sic] (OBERF)

I was 24 when my wife died in a car accident. A month later, my friend was brutally murdered. These events resulted in my becoming sad and depressed. The terms "heart broken" and "having the rug pulled out from under you" were true statements of my everyday existence. One morning, regretting having to get up, I stirred in bed, not yet opening my eyes to face another day. My mind was racing again with questions, reasoning and doubts. I had these intense feelings of guilt and agony. The vicious daily cycle was once again about to repeat itself. In desperation, I felt my heart cry out to God for help. He answered. What then transpired is hard to describe in words. I suddenly felt detached from my body and the anguish. The sounds of life outside faded away into silence. I found myself to be floating in a calm, dimly lit, featureless void. Then I heard this loving, calming "voice" say "yes?". This voice was not audible, but it seemed that way. It was more like it was within my head and a part of the void all around me. With the simple statement came an understanding that I could ask whatever I wanted, that it would be answered. That it was here to help me understand and to comfort me. I did not feel afraid. I asked about the most vexing question within my heart. It answered not only in word but in visions. It did this with every question I had to ask; a simple reply in word, with visions and a complete understanding. There was also a growing intensity or vibration with each answer and question, building one upon another. I continued to the point that I knew would be the last question. The ultimate question. The question that answered all questions. It is the only one that I would remember from this whole experience. "but, why?" The answer was just as simple. It answered "love". At the mention of the word, I felt transported into an immense dark void. I felt like a spectator viewing this light growing within it. As it grew, the intense feeling within my soul, which already felt that it could not retain the energy it felt within every fiber of my being, was growing even more along with it. Beyond anything I had ever felt or experienced in my life. I had become one with the light; a part of it, and yet seeming to be with all of it. I was either getting closer to it or it was growing closer to me. It was pure. It was brilliantly white, composed of many lights. It was love. Suddenly, It exploded, nova like, out into all directions. Like ripples in a pond, in waves it went out into the the vast dark void. I felt carried along outward along with it. The incredible love and joy that I felt. Words are inadequate to describe it. I then seemed to smack back into my body. It was almost audible, but without pain. I knew I was back. I heard birds singing outside. Life was continuing on, but it was new. I don't recall ever hearing birds so cheerful, either before or since. I was different. The gloom and despair were gone, not even a lingering memory. It was replaced with this loving, joyful feeling. **My soul was at peace. I did not want to open my eyes, thinking it would all go away with the light of day. The day was one of the most beautiful of my life. I felt centered, living in the "here and now" present moment of time, not in the past or in fear of the future**. I felt "clear", not muddle headed. **The love and joy continued within my heart and soul for weeks. At the time, I felt that I was talking to God**. I now believe that this encounter was with a ministering angel, sent by Him. It makes me wonder even more about Him, this living God who answers prayers. It made me want to get to know Him even more. Several years after this, I became a Christian. **Prior to this experience, I was looking into the spiritual and religious aspects of life. I believed there was a God**. Possibly the same God of the Buddhists, Islamic, etc. I thought that by living a "Good" life was all that was needed. It requires more than this. Our meaning and purpose in life is more than this. I found that the only way to find this source of love I had experienced, and know that it was one and the same, was via Christianity, and not any of the other ways. I have found my way back, and have had a even greater experience than this by His grace and mercy. I also found that He had never left. His Spirit was waiting for me all along. He is waiting for you. Make it your hearts cry. You'll never be the same, and that's a "good" thing. [sic] (OBERF)

# ^27^ There are examples of OBEs that are characterized by neutral or negative emotions:

About four years ago I was napping at a boyfriends house when I felt my mind wake up but my body could not move, I was terrified. I couldn't scream or do anything. I remember there being a presence of something that seemed shadowlike and I was afraid. I initially tried to convince myself that this was not really happening. I finally stopped trying to scream and realized that I was concentrating on my body very closely, listening to my breathing and my heartbeat. The next thing I remember was feeling the sensation snap back into my body. **My initial reaction to this was one of terror**. [sic] (OBERF)

About one hour after I prepared dinner, about 8:00, I went to bed to go to sleep. I had been having trouble sleeping ever since my grandmother passed away. Finally about 8:30 I felt myself going into a deep sleep. Then it seemed like instantly I awoke. My eyes opened. I looked around. I was talking to myself the whole time. I was saying stuff like, there is my chair, there is the bird cage, there is my husbands pants on the chair, I can hear the TV, and I can hear my husband in the living room. I was totally aware of my surroundings, but yet something was very strange. Only my mind was aware of what was going on. It was like I did not have a body at all. I never once acknowledged that I was a person or had a body, just mind. I was totally aware of my surroundings and that was all. I had an overall different feeling than ever before. I had no pain and I was totally free from everything, but yet I knew something was wrong. When I suddenly realized something unexplainable was happening to me, I came to the conclusion that I was dead or dying. **I realized my body at that time, I couldn't feel it or even move and realized I was not breathing, but yet I was still alive and aware of all my surroundings, then in my mind I started to panic. When I stared to panic it was like my mind screaming and I had no control of my mind connecting to my body**. Anyway, suddenly like a tremendous heavy thump I felt this pressure on my heart like never before, and my heart starting beating out of my body. Suddenly, and faintly, **I screamed for my husband to come and help me.** I felt like I left my body and then came back into it. My husband called 911 and when they arrived, I could not even sit up in bed. I was so weak, and pale, and even talking was difficult for me to do. I just knew I was having a heart-attack. [sic] (OBERF)

**About two months ago I had another terrifying one**. My husband and I were in bed for the night. I became aware that I was slipping out, and consciously chose to go with it. This time I went up through the closet and into the attic. I was a bit giddy, though not afraid, and decided to return to my body. I did and attempted to wake my husband. I couldn't make any sound even though I felt myself to be yelling at him. I decided to see what would happen if I let go again. I again went up through the ceiling and into the attic, further this time. **Again I felt silly and giddy and wanted to return. When I got back in my body I again screamed for my husband to wake up**...he rolled over on top of me...but I knew he wasn't awake and couldn't have heard me. He forced his knees between my thighs and pushed my legs open. I looked into his face...it was my husband but his hair stood on end, his eyebrows were bushy and he had a horrifying leer on his face. He said "oh, so you want to get CLOSE to me, eh?" I snapped back into my face-down body and began to scream in earnest until I was able to command my body and actually made enough sound to really wake him up. I told him what had happened, he spooned me and we went back to sleep. [sic] (OBERF)

Around the year 1986, following the time of my flying dream episodes, where I would consciously dream of flying, another peculiar thing happened. I would experience feelings of floating up and also of falling down- as if being dropped from great height. I'd fall and there would be a great commotion upon landing, my physical body shaking vigorously and abruptly for one or several seconds. One time the shaking upon landing was so vigorous that I was sore for at least the entire day. Sometimes the fall would be at the end of a dream in which I fell from a building, or off of a cliff, but that didn't explain the floating sensation. When the most memorable part happened, the falling as if from a great height, I just left it as the enigma it was at the time. This soon changed. One morning in particular I was enjoying this floating sensation, yet with more awareness than usual. Without giving much thought to the fact that I didn't see any of the scenery, or experience any of the movement control usually associated with my flying, I just assumed and accepted that the floating feeling was just another one of my flying dreams, and I went with the flow. At a certain point of lifting I stopped rising and heard an unusual sound. It resembled the sound of a helicopter propeller except it went slower- Whoosh, whoosh, whoosh the sound went- over and over again. I was floating stationary, with this whooshing sound happening around me and through me at the same time, and out of what was maybe curiosity, (and to make sure that I wasn't in any danger); I decided to see what was going on. I opened my eyes to see that I was at the ceiling. The ceiling was only a few inches away from my nose, directly in front of my face. I looked to the right to see the source of that sound. The ceiling fan was on, and its blades were going directly through my body! In shock I turned my head downward to see a lump on the bed, covered in sheets. My physical body! I was floating mid air, only a few inches from the ceiling, and in a body that felt as real as my physical body! Yet my physical body was still on the bed, lying asleep! **I panicked- and rightfully so I'd say.** I felt myself take a large gasp of air, and I instantly fell back down to my physical body, with a cluster of fierce jerks throughout my body upon landing. As I lay there I pondered in awe, and from that point on I knew what was happening when I fell. I actually fell back into my physical body! [sic] (OBERF)

As a young woman in my twenties I was struggling to find my purpose and my identity. I was living with a friend in an apartment and working overnight shifts at a local pharmacy. I had recently come out of a deep depression from a relationship that ended and broke my heart, but was working to get back on course with my life. It was a difficult time for me but my job kept me focused and moving forward. On the day of the incident, I came home as usual and went to bed to sleep and set my alarm to get up again for work later that night. I typically went to work 10pm-7am so would sleep from around 9 or 10am to 6 or 7pm. I slept soundly that day, until I suddenly awoke to the sensation that I was moving. I suddenly found my 'self' (spirit) being pulled backward into my mattress, like I was falling backward but in a controlled way. I did not open my eyes. I could feel the inside of the mattress but not with my physical 'self'. I could feel the foam and the springs! I didn't know what was happening and was freaked out! Suddenly I felt like I was on a rubber band and gently stretched back, then flung out of my body. I still did not open my eyes. I could then feel my 'self' floating above my body, facing the ceiling. Still I did not open my eyes. I could sense the space between my body and my 'floating self' I had the sensation that my hair was hanging back from my head as I floated there, in my bedroom above my body and bed. I was both scared and intrigued. This all happened in a matter of minutes. I think I didn't want to see; I was afraid. Suddenly I found myself no longer in my bedroom, but roughly 10 miles away in my parent's bedroom in the house I grew up in! I was fully conscious and now my 'eyes' were open. Except I had no body! I was just a single, tiny pin point of perspective floating there. I could see 360 degrees around myself all at once! **I was still frightened but also intrigued.** Their bedroom was dark and my parents were not present, however this would have been during their usual dinner time if this event occurred in 'real Earth time'. I was facing their bed. To my left I saw 3, maybe 4, people - shadowy figures- just observing me. I could not make out their faces but could sense bright eyes watching me silently. I got the feeling that they were very somber people and weren't permitted to speak to me but only watch. My attention was suddenly to my right however, as a large bright white figure of light stood before me. It was made up of all light, also had no face that I could make out. It stretched from ceiling to floor and appeared to somehow be bigger than the room yet still contained within it. It was shaped like a man wearing long draping clothes but really it was all made of light. At the top of its 'light head' was something shaped like a man's top hat. **I was again very scared but also intrigued**. I felt so small. I then found my voice and spoke, I said, ' WHO ARE YOU?!' and I was surprised at the sound of my voice. I was much more assertive and demanding than I normally was at that time of my life. The being of light replied to me in a loud, stern, clear voice 'You KNOW who I AM.' I most certainly did not, that was why I was asking. I was frustrated because he would not identify himself. Overall, the room felt sad. The people to my left just hovered there not speaking, I noticed that the window above my parent's bed showed there was some grayish light outside, which didn't make sense since it would have been nighttime and dark that time of year. I was not satisfied with his first answer so demanded again, 'Who ARE you???!' I was scared and therefore defensive. I felt like I had to 'stand my ground' (although I had no feet!) Again, the being of light said sternly (and maybe with a little annoyance?) 'YOU KNOW WHO I AM.' At this point I had some ideas, some speculations, some guesses, but did not believe myself. I couldn't wrap my mind around what was happening. **All of a sudden I was flying, rushing very fast through a tunnel of light and I was screaming 'NOOOOO!!' because I was terrified and had no control over any of this.** Seconds later I was SLAMMED back into my body. I did not view my body, I just felt my 'self' slammed very hard backside-first into it, so hard that once back in I sat straight up in my bed bed , with my physical body, and was still screaming 'NOOOOO!'. After a few moments I calmed myself down and reviewed what just occurred. It shook me up. [sic] (OBERF)

# ^28^ The following examples demonstrate the emotional quality and positive after-effects associated with LD:

While ascending a mountain path I began to find it more and more difficult to climb. My legs took on the familiar leaden feeling they sometimes have in dreams, and a dull heaviness spread through my rapidly weakening body. My feelings of weariness deepened relentlessly until I could only continue by crawling—but finally even this was too much for me and I was overcome with the feeling of certainty that I was about to die of exhaustion. This realization of imminent death focused my attention with remarkable clarity upon what I wanted to express with the one act of my life I had left: perfect acceptance. **Thus, gladly embracing death, I let go completely of my last breath, when to my amazement and delight a rainbow flowed out of my heart and I awoke from the dream**. **Years after this experience, the profound impact of this dream of death and transcendence continues to influence my beliefs concerning what may happen to us when we die.** (LaBerge 1986)

With the realization of this fact [to be dreaming], the quality of the dream changed in a manner very difficult to convey to one who has not had this experience. Instantly the vividness of life increased a hundredfold. Never had sea and sky and trees shone with such glamorous beauty; even the commonplace houses seemed alive and mystically beautiful. **Never had I felt so absolutely well, so clear-brained, so divinely powerful, so inexpressibly free. The sensation was exquisite beyond words** but it lasted only a few moments, and I awoke. As I was to learn later, **my mental control had been overwhelmed by my emotions**. [sic] (Green 1968)

I am able to dream lucidly on occasion which means becoming aware that you are dreaming while you are asleep, and continuing on with the dream with full consciousness. Usually I just fly in my lucid dreams which is incredibly enjoyable. This time, the moment I 'woke up' in my dream, I had an overwhelming sense of opportunity that I had to grab right away. I said out loud 'I want to be free!' And the intention was 100% to experience real freedom from all my earthly bonds. The desire to do this came out of nowhere and was complete, and I knew I could do it in this lucid dream, though I didn't know what it would mean to do it. Immediately I was taken. I was simply swept up by a force that took me away at great speed. It was as though this force was just waiting for my request, totally ready to help. I felt like I was inside this force, like being held. It was not good or bad, it seemed somewhat neutral. I had no control. I was entirely lucid and could feel everything. It was very surprising to be swept up like this. The force took me through the clouds and sky and then beyond. I realized we were leaving the atmosphere. I like to fly very high in my lucid dreams, but this was farther than I'd ever gone. We kept going and soon we were heading out into deep space. I realized we were leaving the Earth entirely and I thought that I must be dying. I was so lucid and aware of my body, it was all so real, that I was certain this had to be death - that it would be if I just let go. I 'turned around' so to speak and looked back. The Earth was hanging in space, so far away now, about the size of a large marble. **It was beautiful. I looked at it with love and I felt a total joy and sense of gratitude for my life on Earth and everyone and everything in it - even the bad people and experiences. It all felt perfect and absolutely correct. My sense was that I wanted to clap and say 'well done!' to everyone**. I had no desire to return. So we kept going. The force took me through the universe. I traveled through galaxies and stars. I was moving at an incredible speed. Eventually I came up to what seemed to be a void. There were no more stars. There was a blackness in front of me. A sound emanated from it that I can't really describe. Sort of a droning hum. I didn't really like this black void. It didn't feel hostile as much as inhospitable to me. I couldn't enter it with my body, which I still seemed to have. I felt I couldn't go in there and I didn't want to anyway. I looked back over my right shoulder, behind me, and there was a vast ball of churning light full of what looked like neural pathways. I knew it was the Universe in its entirety. I was way beyond the outer limit of where life and energy was. In front of me was the Void. I decided I needed to go back. The absolute second I decided that, I was whooshed away right back the way I came from. We travelled all the way back until Earth appeared and I zoomed down through all the layers until I landed in my room, in my bed, in my body, with a physical Thump, and my eyes immediately opened. As I had been descending I felt sorry for what I was about to take back on again - the weight of this life. I was sad when I woke up again in this body, but also amazed and grateful for the experience, which felt entirely real to me. **I hope that when death does come I feel that same gratitude and sense that everything was just as it should have been in this life.** [sic] (OBERF)

After a few weeks of intense anxiety, I decided to schedule a night to perform wakeups to help induce a lucid dream with the plan to connect with my inner child. After the first wake up at 3:30 am I happened to look over at my chest of drawers, where Blossom the pony (a childhood toy) had been sitting since my mum found her a month prior. Instinctually I nipped out of bed and collected her, and snuggled back under the duvet with her held in one hand. Focusing on my breathing and the sensations of Blossom in my hand, I eventually transitioned through the hypnagogic and into a lucid dream. **It took a while but after calling out several times and resorting to my childhood nickname (Treenie-trops!!) there she suddenly was, crying and alone and in desperate need of some love, reassurance and connection. I walked over to her - she was so tiny staring up at me and calling out for mum - hugged her tightly and told her I loved her. I felt much more at peace in the following days and it definitely helped shift some blocked energy**. I have since used this technique again, holding a rock of significance when I went to bed (without doing wakeups). In an unconscious dream I had I was holding onto a rock and it fell out of my hand over a cliff. The unfolding of events this resulted in me becoming lucid. When I woke up out of that dream into waking reality, I was no longer holding the rock, so I wonder if this translated within the dream. [sic] (LDE)

After falling asleep I woke up in my dream. I found myself facing some obstacles where I have to pass through some narrow passages and I manage to do so. I found my self at a burning hill and I couldn’t go up the hill. Then someone appeared and somehow I knew that he was my master. As he got close to me I asked him, “Are you my master?” He nodded his head yes! An inner voice came to me and said, “The master appears when the student is ready.” Wham! I got it! I held him in a big hug and burst into tears. I cried and cried, thanking him. Then I looked at him. He was an old Indian man. I asked him, “Are we in India?” He said yes! Then he showed me an ancient city - like my eyes were opened to see it. It was so beautiful, like it was from an enchanted magic story. Then I saw people from all over the world coming to listen to what we had to say, and go into the city. The interesting part is that I was fully awake in my dream to what was happening. **Then I woke up in this reality. I had tears still on my face. I was actually crying for real. This dream taught me to build self-confidence to get to my goal no matter what obstacles are encountered in life, or how narrow and scary the path. I interpreted the Master appearing as my own self; becoming my own self, my own Master. It has given me the strength I need to confront this reality**. Namaste! [sic] (LDE)

# ^29^ There are instances of OBE stories where the individuals do not report literally seeing their physical bodies or have altered perceptions of their physical bodies during the experience:

A set of what seemed to be personal synchronicities led me to a moment of complete surrender and I asked to go home. I felt something within my being rise up through my chest into my throat. I remember at that time sensing a voice and hearing , relax everything is ok. In the next instance I heard this immense crack in my sinus area, with my head slightly tilted back and mouth open, some part of me reached out or left my physical body, through my mouth. In what seemed to be an instant I found myself in the middle of some sort of arena with a overwhelming feeling of love and an equally strong feeling of gratitude for all and everything that I had experienced in that/this life. I noticed in the darkness some figures in hooded robes, no bodily feature could be seen. As I realized some sort of connection had been felt and I had no feelings of inhibition, I opened up with a free flowing feeling of love. As I was in this moment I noticed more being appearing and participating in whatever was occurring. Whatever took place in those moments is and has been hard to explain. I got to ask one question and that was, who are you. A voice that I will never forget said I Am. My first response was, Who is I Am, What is I Am and then I felt myself leaving, kicking & screaming. When I regained some of my senses I thought, who could I tell and in my imagination I thought words are to limiting & what does it mean. [sic] (OBERF)

About 16 years ago, my girlfriend, I and our three boys spent a weekend together camping. The first evening, there, we all sat around the campfire roosting marshmallows and chatting. After the boys finally went to sleep in their tents, my girlfriend and I continued chatting for an hour or so before bedding down in our camper van, at about 1-1:30 am. Due to my girlfriends loud snoring I had difficulties falling asleep. After some time, I decided I have better start willing myself to think of anything but her snoring. I do not recall how much time passed between the time I heard the snoring to the time I felt something or some force trying to leave my body. The first thing I recall was, this indescribable feeling of something wanting to leave my body. The feeling was mostly concentrated in the center of my chest, I have no idea as to the length of time this feeling lasted. In any case, this feeling needed to be released and for whatever reason, it needed me to open the top of my head in order to be released. I thought it quite remarkable because if it was coming through my mouth I would for sure vomit. What seamed like a fog or grey mass, slowly began floating upwards in a perfect straight line and continued floating upwards and out through the top of my head. Once released, I was filled with love, laughter and tears all at the same time. I remember, thinking, and telling myself, NOT to open my eyes or I might loose 'sight' of this wonderful feeling. I looked up, my eyes still closed, and there - just above the temple of my head - stood this incredible entity of pure light in the shape of an Engle, gesturing me to follow. No words were spoken I just understood. I remember not being able to determine if the entity was male or female. It was not a solid mass, rather a shape in various shades of white --illuminating itself. Something similar to viewing a laser light-show, except this light overflowed with warmth and love. Before I knew what happened, I was in total darkness. I opened my eyes, this time for real. Before me was earth, as in the whole earth. My first thought, Oh my god, I can see the whole earth, how small and vulnerable it looks. I don't remember seeing any stars or other planets only earth. Next, I was asked; Where do you wish to go? What do you mean where to go, I asked? Thinking, I wouldn't mind going to my country of birth if only to see my father, telling myself, it's too far and it will take too long and, silly me, what am I thinking, my father passed away long ago. It wasn't till then, I realized I was floating above earth, asking myself, how is that possible? Thinking, I am going to fall down. I was told I was quite safe and in case I was lost I had a lifeline reaching from earth to my umbilical cord. I looked down towards my navel and sure enough there was this extraordinary thin cord like only a spider could weave. **I looked down at myself again. I didn't recognize my body, there were no legs or feet, certainly no clothe. It didn't alarm me nor did I feel differently, I just looked differently. I felt like I had a body, but it didn't look like a body.** At the time, none of that seam to bother me. My attention turned again to the umbilical cord, my eyes following the length of it down towards Earth. The closer to earth the cord was the more it glowed and shimmered like a silver cord, connecting me to earth. I started having doubts about this cord. What if it broke, how was it possible for this cord to keep me up here? What if I couldn't find my body? In an instant, I was back in my body, with a force so hard I bounced back up, hitting my head on the ceiling, totally forgetting I was in the top bunk-bed of a van with a clearing of about three feet. I have never voluntarily told this story to other than the friend accompanying me on the camping trip. Whenever the subject arises I only speak of it briefly. I understand and appreciate people having difficulties believing it was other than a dream. My only wish is that, one day, I may successfully, re-live this extraordinary feeling of love and trust. Believe me, it is not for the lack of trying. [sic] (OBERF)

About 6 times in two months, when in bed trying to sleep, felt my body suddenly paralyzed and a strong frightening noise in my ears. Tried to move and wake my partner besides me but completely unable. Then a loud "pop" sound, feeling like flesh ripped of my body and suddenly legs floating, like pulled up by something and then rest of body out. Each time find myself exactly room in front of the bed where I lay down (sometimes my own, sometimes my daughter's, sometimes the sofa). Seeing each time the furniture and objects around like lit from inside (by themselves) I felt everything had its own light and sound as well. Tried to move, just thinking about going down the corridor and then being "sucked" to the place at an amazing speed. Feelings of lightness, I could think incredibly clearly, everything seemed much more real than reality. Sort of concepts or information about life and its purpose coming to my mind. Everything around recognizable but with funny distortions. Sometimes some object or shape I wouldn't recognize. **I could not see clearly my body lying down but could feel the shape somewhere there.** I could perceive though the body of my partner bed. Then suddenly back and sit up in my bed sweating, not understanding what happened, quite frightened to go back to sleep for some days. It's happened about six seven times since then and not so frightening anymore though I can't control the process voluntarily. Also find that depending on my previous emotional state or types of thought the experience can be pleasing or terrifying. Most striking the quality and clarity of consciousness and the vividness of feeling. When it happens it seems as "this reality" is a dream. And you don't find important the things that you usually care about during the day. [sic] (OBERF)

After a long day I was happy to retire to cozy bed for a good nights rest. I was alone my husband was on night shift. I was lying on my back covered up (on a cool night) and head resting on my pillow feeling myself falling asleep...then with my eyes closed half into a deep sleep I became alert to the loud sound of a screeching roller coaster. The wheels rattling and roaring sound you hear from a downward fall from a high hill on a wooden roller coaster. Then I lifted my head and looked at my body (with eyes still shut) and thought this is strange. Then I saw two views, one of my clear blue arm and head rolling to the right out of my body. This view was from above. Then the other view was as I was rolling out my body seeing it from real-time, in-motion...I noticed my body sleeping with slobber on the side of my mouth (mind you I drool when I am in a deep sleep)... then I find myself floating in the corner of my bedroom. I see my body on the bed realize that I'm not in that body and feel curious as to what is going on. I turn around face the top of my bedroom door and put my hand through the wall look back at my body and the next thing I remember was waking up thinking what in the world just happened**. One thing to note that I found interesting was when I saw my clear blue arm and head roll out of my body I noticed I had the outline of a young man's muscular arm. I find it strange because I am a woman. Also the color of my body was a light blue see-through almost like if you look at a clear see-through light blue balloon**. [sic] (OBERF)

# Not seeing one’s own body is a feature also observed in many of Robert Monroe’s stories. In several instances, he details his OBEs as “visits” to family members and friends and does not explicitly mention any awareness about his physical body:

I floated upward, with the intent of visiting Dr. Bradshaw and his wife. Realizing that Dr. Bradshaw was ill in bed with a cold, I thought I would visit him in the bedroom, which was a room I had not seen in his house and if I could describe it later, could thus document my visit. Again came the turning in air, the dive into the tunnel, and this time the sensation of going uphill (Dr. and Mrs, Bradshaw live in a house some five miles from my office, up a hill). I was over trees and there was a light sky above. Momentarily, I saw (in the sky?) a figure of a rounded human form, seemingly dressed in robes and a headpiece on his head (an oriental concept remains), sitting, arms in lap, perhaps cross-legged a la Buddha; then it faded. I don't know the meaning of this. After a while, the uphill travel became difficult, and I had the feeling that the energy was leaving, and I felt I wouldn't make it. With this thought, an amazing thing happened. It felt precisely as if someone had placed a hand under each arm and lifted me. I felt a surge of lifting power, and I rushed quickly up the hill. Then I came upon Dr. and Mrs. Bradshow. They were outside the house, and for a moment I was confused, as I had reached them before I got to the house. I didn't understand this because Dr. Bradshaw was supposed to be in bed. Dr. Bradshaw was dressed in light overcoat and hat, his wife in a dark coat and all dark clothes. They were coming toward me, so I stopped. They seemed in good spirits, and walked past me unseeing, in the direction of a smaller building, like a garage, Brad trailing behind as they walked. I floated around in front of them, waving, trying to get their attention without result. Then without turning his head, I thought I heard Dr. Bradshaw say to me, "Well, I see you don't need help anymore." Thinking I had made contact, I dove back into the ground (?), and returned to the office, rotated into the body and opened my eyes. Everything was just as I had left it. The vibration was still present, but I felt I had enough for one day. (Monroe 2014)

As I relaxed, the vibrations came and then an impression of movement. Shortly thereafter, I stopped, and the first thing 1 saw was a boy walking along and tossing a baseball in the air and catching it. A quick shift, and I saw a man trying to put something into the back seat of a car, a large sedan. The thing was an awkward-looking device that I interpreted to be a small car with wheels and electric motor. The man twisted and turned the device and finally got it into the back seat of the car and slammed the door. Another quick shift, and I was standing beside a table. There were people sitting around the table, and dishes covered it. One person was dealing what looked like large white playing cards around to the others at the table. I thought it strange to play cards at a table so covered with dishes, and wondered about the overlarge size and whiteness of the cards. Another quick shift, and I was over city streets, about five hundred feet high, looking for "home". Then I spotted the radio tower, and remembered that the motel was close to the tower, and almost instantly I was back in my body. I sat up and looked around. Everything seemed normal. (Monroe 2014)

# ^30^ In the context of LD, it is not uncommon for subjects to refer to their physical bodies:

In the night of January 19-20, I dreamt that I was lying in the garden before the windows of my study, and saw the eyes of my dog through the glass pane. I was lying on my chest and observing the dog very keenly. **At the same time, however, I knew with perfect certainty that I was dreaming and lying on my back in my bed**. And then I resolved to wake up slowly and carefully and observe how my sensation of lying on my chest would change into the sensation of lying on my back. And so I did, slowly and deliberately, and the transition--which I have since undergone many times--is most wonderful. It is like the feeling of slipping from one body into another, and there is distinctly a double recollection of the two bodies. I remembered what I felt in my dream, lying on my chest; but returning into the day-life, **I remembered also that my physical body had been quietly lying on its back all the while**. This observation of a double memory I have had many times since. It is so indubitable that it leads almost unavoidably to the conception of a dream-body.[…] (Van Eeden 1913)

I dreamed one night that I was floating in the air above the dark ocean. **I knew myself to be roughly a hundred miles to the N.N.E. of my boat's present position**. There on the ocean, on the dark water below me, I saw a curious hull which I took to be a derelict. As I looked at it, the light increased and I made it out to be a ship with a stumpy mast and the Captain's bridge aft resembling a hen-coop. [...] (case 61) (Crookall 1966)

Without any preliminary ordinary dream experience, I suddenly found myself on a fairly large boat travelling at a normal speed up what appeared to be the mouth of a river, just before it issues into the sea. There was some sort of pleasant scenery on either side, with trees and greenery, and straight in front, the water stretched to infinity. The deck was smooth and clean and warmed by the sun, and I felt the warm breeze on my skin. This startled me, because I knew that in a dream one does not feel actual physical sensations with the same intensity and subtlety as in real life, and I was sufficiently mistress of my own thoughts and movements to pinch my arm in order to assure myself that it was only a dream. I felt the flesh under my fingers and the slight pain in my arm, and this filled me with real alarm, because I knew that I ought not to be on that boat, in the daylight. **I did not see my own body, but I was sufficiently lucid to imagine it, lying inert in my own bed here in Paris** […] (Green 1968)

# ^31^ The use of bizarreness to distinguish between OBEs and LDs is fraught with issues, as the interpretation of the experience varies among different individuals. While some subjects may interpret bizarre experiences as an OBE, others may perceive it as a LD:

[…] Miss Peters made critical observations: opening a drawer she found everything disarranged, whereas it had been left in good order. **The incongruities between the physical and non-physical environments made her realize that 'things were not normal' and that she was outside her body**. (case 48) (Crookall 1966)

[…] **I saw a man who seemed normal except that his head appeared to be composed of yellowish gas. This dream-like character struck me, and I said, ‘This is a dream!’ As soon as I did this, I became fully conscious and found myself "projected".** The dream had changed into reality! (case 117) (Crookall 1966)

[…] I’m sitting in the car outside a store. The lights, goggles go on. I feel them on my face. I wait for them to turn off before doing a reality check. I reach up to take the goggles off... then the goggles aren’t there anymore and, still sitting in the van, **I decide to test reality by reading a dollar bill. A word is wrong, so I conclude I am dreaming!** I get out and fly. It feels wonderful. The streets are bright and sunny, crisp and clear. I fly up over a building and the sun gets in my eyes-it is the light washes out the imagery, so I spin my body. I end up inside the store with friends, no longer lucid and tell them about my experience.” (LaBerge and Rheingold 1997)

[…] I was in Minneapolis on a sunny, early spring day. A foot of snow lay on the ground. I am standing at the bottom steps of a porch, while on the porch are four other people. **Covering the porch steps are hundreds of amber and emerald gems and crystals. This seems too odd, which triggers the realization that I'm dreaming.** (Waggoner 2008)

At the mall with friends. One of them is a good looking young guy with dark hair. We all get separated and I run across the guy again. We talk. He is well dressed. I feel envious of his style and good looks. Sense hostility from him. At the metro I see Rene waving at me from a distance, amid the crowd. He seems very friendly. I try to reach him but lose him. I reach a large platform which is less crowded. A former female high school colleague stands there, looks older. I see a familiar looking boy in a train, get into the train. Upon looking closely, I realize it's me as a kid. The young me is sitting beside a girl and a cat. This girl looks exactly like my mother but the same age as the young me. They are playing happily. The cat is white and orange, holds a sign in its mouth with a year from the 90s, can't remember, possibly 1992 or 1993. I hear my adult mother's voice coming from the cat. They are supervised by one or more adults whose faces I don't pay attention to, but voices sound familiar. **Everything looks extremely vivid but the situation is absurd; I realize I'm dreaming**. For a very brief moment I try to approach the children and the cat. The cat's eyes are of a bright pink color. The pink hues move in a spiral, hypnotic pattern. Suddenly it has only one eye. I'm mesmerized by everything and try to stay in the dream but everything turns dark very briefly after my gaining lucidity and I wake up. [sic] (DreamJournal)

# ^32^ The following anecdote illustrates that the term “dream” is commonly used to describe experiences perceived to have occurred in a non-physical realm, often regarded as “not real”, while the term OBE is typically associated with experiences that are believed to have taken place in the physical world, commonly seen as “real”:

I did not do anything to bring on the experience and had never considered anything like this happening to me. But one day, about 5:30 in the afternoon, it happened. I had just gotten off work and I was exhausted. I was back staying with my parents after a divorce from my high school sweetheart. My mother was the only other person there. I had my work clothes on and a pair of heavy- duty work boots. I walked into the extra room and sat on the Murphy bed. was so tired that I laid back and stretched out like someone going to sleep. I had no intention of actually sleeping nor did I do any more than close my eyes for a few seconds. I was very conscious and alert. Even though I had been tired I was hyper-alert during the experience. The experience happened in bed but it was not dream-like in any way. Except maybe the reality challenging aspect of it. **There is no way, that what happened to me could be a dream. It is true that all dreams seem real to us, while we are having them. However, the instant you wake up you realize that it was a dream. What happened next was no dream.** I felt something grip my ankles. It felt like hands squeezing and lifting my heals. I say hands because I felt the cupping, from the back of the heals, and the thumb across the front of the ankle. Anyway, it was lifting my boots, not my feet. I now find that strange. I would think a floating experience would involve your body, not your clothes. Anyway, something lifted my feet, as a person would do if they were attempting to slip off your shoes. […] But I sure didn't figure on what happened next. **Believe me, if I was making this up, I sure wouldn't include something as unbelievable as the next part. But I am telling all, and telling it like it was**. The hands did not go away. They lifted my heals back up again. Then they paused for a second and gripped me tighter. Then they snapped me. […] I looked up and saw two almond shaped openings with light coming from them. I didn't realize it at the time but in thinking back, the heavy vessel must have been my body and the almond shaped openings, my eyes. **Until that day, I had always thought of my body as myself. I had not experienced a separation of my consciousness in anyway**. I was confused at first, I was just trying to figure out what was happening to me. All I knew was that when I dove towards the openings, I was sucked right through. I floated up and remember the freedom from gravity. […] That is when I looked down at my body. I didn't notice any "silver cord" connecting me to my body, like some people claim. […] But when I looked down at my body, I thought I was dead. Extreme fear hit me and I panicked. I dove back into the eyes and was suddenly safely back in my body. […] [sic] (OBERF)

# ^33^ There are several examples of stories where the disembodied element is present but interpreted as a dream rather than an OBE:

In nearly all of the nearly nine hundred lucid dreams that I have recorded, I have been embodied in the dream in the accustomed guise of myself. In only three cases was I playing a role other than "Stephen LaBerge" when I realized I was dreaming. **The exceptions are interesting: in one I dreamed I was simply a disembodied point of light.** (LaBerge 1986)

I would like to describe one of my own wake-within-REM-initiated lucid dreams. It was the middle of the night, and I had evidently just awakened from a REM period since I effortlessly recalled a dream. I was lying face down in bed, drowsily reviewing the story of my dream, when suddenly I experienced a very curious sensation of tingling and heaviness in my arms. They became so heavy, in fact, that one of them seemed to melt over the side of the bed! I recognized this distortion of my body image as a sign that I was reentering REM sleep. As I relaxed more deeply, I felt my entire body become paralyzed, although I could still seem to feel its position in bed. I reasoned that this feeling was most likely a memory image and that actual sensory input was cut off just as much as motor output was. I was, in short, asleep. **At this point, I imagined raising my arm and experienced this imagined movement as if I had separated an equally real arm from the physical one I knew to be paralyzed. Then, with a similar imagined movement, I "rolled" out of my physical body entirely.** I was now, according to my understanding, wholly in a dream body in a dream of my bedroom. **The body I had seemed to leave, and which I now dreamed I saw lying on the bed**, I quite lucidly realized to be a dream representation of my physical body; indeed, it evaporated as soon as I put my attention elsewhere. My brain's representation of my body image was no longer constrained by sensory information concerning my body's actual orientation in physical space and I was free to move it in mental space to any new position that I chose. With no sensory input to contradict me, I could freely "travel" anywhere in mental space. (LaBerge 1986)

**I begin falling into a dream**. **I quickly realize I'm starting to dream and phase back to my body**. I'm immediately put into the vibrational stage. **I start floating out of my room as a disembodied point of awareness with no body**. I float out down the hallway to the kitchen and through the backdoor. I'm floating in the driveway now. Everything is very vague and fuzzy. **I find myself back in my body. I start spinning my astral body**. I start hearing a weird insect-like buzzing noise when I do this. The vibrations grow stronger. I find myself in a dark, void-like space. I feel a strong vibrating energy in my lower left abdomen. I've felt this many times before when in the vibrational state, and when I did DMT. I start to see little white specs and orbs of light blink in and out of the space around me. I put out my hands in front of me. A little white orb of energy comes out of my finger. I do this a couple of times, creating more orbs. They stay for a few seconds and then disappear. The dark void space i'm in starts feeling like a deep, deep part of the ocean. There's no water, but the air is thick and there is utter blackness around me. I have the feeling that there are predatory beast like things swimming around me. I have a feeling of hopelessness and fear in this space. There's an intuitive feeling that this is a space that some unfortunate souls can find themselves in after death. I feel sorry for anyone in this space who is confused enough to stay in it for longer than a few minutes. It's so dark and lonely. It seems like a type of hell realm, although obviously not permanent. […] As much as I want to explore more, and as much as I know the tincture I was just given will probably allow me to stay here for who knows how long, I decide to wake myself up to write all this down in order to preserve the memory. (DreamJournal)

[…] All at once I knew that I was dreaming ... **It was then that I became aware of my spirit, that is, that part of me I think of as my "self," detaching itself from my body and floating across to his body. In this bodiless state** I was able to use all my senses to orient myself, that is, seeing, hearing, feeling, etc. **After I had left my body**, I still saw it standing there, doing some sort of fiddly work and talking. In other words, you could not tell by looking at my body from the outside that I was no longer inside it. So I floated across to the boy and slipped in his body. As I did so, I had the feeling that I had taken over all his vital body functions and his motricity ... I saw with his eyes, I saw my body standing there engaged in some sort of activity. I also saw his spirit, his consciousness, I saw him thinking, without being able to remember how this came about . . . I saw how he perceived me, the effect I had on him, and the feelings he had for me [...] (Tholey 1989)

**The dreams I have are very vivid and real**. **When falling asleep I can feel myself drifting away. I get up from my bed and walk to my kids rooms**. Everything is the same, their rooms, my dresser and everything I have on it. I walk back to my room and I can see myself sleeping. The feeling is very uncomfortable for me. I feel afraid and scared of leaving my children behind. I take a look at how the me in bed is positioned and **place myself back into my body**. This is not the first time I've had **dreams like this** one, there have been times I find myself floating through my hallways and pull myself back by placing my hands on the ceiling and pulling. It's a little to weird for me. [sic] (OBERF)

One morning I plunged straight into the **dream state from wakefulness to experience a wake-induced lucid dream (WILD)**. I found myself in a stark interrogation room (think The Matrix). A plain-clothed police detective sat across from me at a metal desk. His monotone voice droned arrogantly concerning some legal matter. I decided to ignore the suit and explore my lucid dream. I had been planning to ask the subconscious mind about a problem at my job in the animation industry, hoping to receive a verbal answer the way some dreamers report happening. **However, before I could begin any dream work, the arm of my “subtle body” popped out of my real arm, pivoting from the elbow**. I gazed at the ghostly hand: it was bluish-white and slightly translucent. I couldn’t tell if it had bones inside or not. This spectral limb jerked from one pose to another—lacking what animators call "breakdowns." Now hinged from my shoulder, the arm swung rigidly behind my back. **I had the distinct feeling that this extra body part, despite its autonomy, belonged to me, so I was not afraid. But it was disconcerting to watch my hand moving with a will of its own.** I wondered what—or who—was motivating this subconscious appendage! Was this a partial, out-of-body experience (OBE)? Once I shrugged off the idea of having two right hands (one real, one astral) I got down to business: I did the Waggoner thing and shouted up at the sky to the awareness behind the dream, asking it to show me the solution to my struggle for productivity at work. No answer. So, I asked again. Presently a rectangular, violet window appeared before my eyes, with a small, convoluted object nestled inside—glowing light purple, with dark contours. I guessed it represented an optic nerve or something in my head. (Upon waking and searching Google Images, I’m now inclined to think it resembled a solitary neuron...or perhaps a cancer cell?) Peeping through the aperture floating before me in the darkness, I scrutinized this complex origami closely: it was quite vivid and three-dimensional (like a fractal surface), and I perceived an hour-glass structure in the walls of the grotto containing it. Was this a cryptic, visual answer to my query? I asked the dream consciousness what it meant, but still received no vocal answer. The rectangle slid upward—it was difficult to keep my eyes trained on it—like a floater adrift on an eyeball. I awoke. The vision persisted for a few seconds in the darkness of the bedroom, slowly fading **as the dream energy dissipated**. Perhaps I was merely seeing hypnagogic imagery, and the purple enigma, locked in its little keyhole, was not necessarily the dream emissary’s answer to my problem...unless I was being shown a brain tumour that needs removing! (LDE)

**After that 'inner' dream, I 'woke up' to another, 'outer' dream**, as I perceived formerly (while later I understood it was an NDE). I saw myself in my room, **I was out of my body**, and was slowly going up. On the right side of my bed is my PC, I saw my little brother working with the PC. Although we couldn't see each other, I was aware that my mother was in the living room and I think I saw here from rood view briefly. I noticed she said something, probably to me little brother (actually, I was not hearing anything). As I was going up, my little brother looked at me and 'said' that, 'hey, you're going up!' while laughing/smiling. I noticed my body below, and it was impressing to me that I was completely indifferent toward it, **I was feeling that it no longer belongs to me, and I was no longer concerned about my body** and the earthly world. I was very peaceful. Then I noticed a white light above near the roof, it was not irritating my sight, and I knew it is God and I'm going to go upper and embraced by the light, i.e. the God. I had a pleasant feeling then. After that, I almost stopped going up more, and a voice, which I don't know if it was my own voice or voice of a third being, said, 'But you didn't performed prayer last night.' I regretted that I didn't performed prayer last night, because it was a shame that the night before going to God, I had not performed prayer appropriately or connected to God. Then, unwillingly, I was sending back downward slowly. **After being back in my body**, I found myself going out of bed, I was going to kitchen, I saw my mother in the living room who was looking at me, I went to kitchen to perform wudu, preparing to perform Islamic prayer in the afternoon (which I wouldn't perform usually). As I was in the kitchen, I saw my father entering the house. After this, I eventually woke up in reality, I saw my little brother is working with the PC, **as I have just witnessed in the dream**. (OBERF)

I was quite tired as I prepared to go to bed. I didn't know why, but I also felt unusually anxious. In bed I read the last chapter of an OBE book I had been reading. Then still feeling a little strange, I turned out the light. As I started to drift off to sleep, **the first images of a dream had already begun. Still awake, I watched as my dream self entered a New Age store. In the dream**, I looked to the left and then to the right before a gentle male figure appeared in front of me. I immediately recognized that he was not part of the dreamscape. He had a more real and solid quality to him, yet I couldn't make out his features. I assumed he was a guide. He extended his hand towards me. I was still awake as I watched him offer me a CD. I understood that the CD was to help me relax, go deeper than I could on my own. **As my dream self** reached to take the CD from his hand**, I immediately began feeling groggy while losing the simultaneous awareness I had of me in bed while also being a dream figure in a New Age store. My awareness shifted to my astral body**. I was laying flat on my back inside my physical body. Then, I began to slip deeper and deeper in a downward and backward motion through what felt like membranes or dimensions. I was moving far beyond the physical world. I realized I could increase the speed of motion by repeatedly relaxing and surrendering deeper. I was fully lucid and aware of what was happening; blissfully absorbed in the luminescent energy of my astral body as I moved at light speed faster and faster, deeper and deeper with each thought of relaxation. I wanted to go as far as I could go, even if I never came back. **But, suddenly I returned to my physical body.** I was still fully aware; never having lost consciousness from the moment I climbed into bed. I lay on my back watching sparkling energy move rapidly in front of my closed eyes. I was filled with gratitude. (LDE)

# ^34^ The stories below show problematic situations in which the terms “dream” and “OBE” are used interchangeably:

**When I exit my body during the dream state, I am fully aware of the fact that I am in some type of dream state. I am outside my body**. I can walk into the living room, create a door and exit outside. I can create images or they just appear before me. All of my senses seem real. I can feel the dampness of grass at my feet when I am walking on my lawn. I can feel the soft fur of my cat that I am petting. I can feel the mist of water on my body from a waterfall, I can hear beautiful music playing. I can increase the volume or lower it by concentration. It is really awesome. (OBERF)

**It was all in the one dream**. I was in my parents back garden and I got this really strange sensation in my body it was not a nice one. Felt like I had no control over what was happening. **Then my body turned on its side so I was facing the ground in a horizontal position at that point I felt myself leave my body**. I felt much lighter and kind of floated into the sky. It was a wonderful sensation, I felt free. **After that I was able to do the obe anytime or anyplace**. **Once in my dream I told my sister that my soul could leave my body. I showed her and she watched me. When my soul returned back into my body I asked her what my soul looked like while floating in the sky she said it was a light. Very strange dream but one that felt very real, especially the sensation in my body just before my "soul" left my body**. It was a sensation that made me feel ill as if the energy around me was being moved. It felt like a sensation I once had while I got an energy rebalancing done from a reiki healer, without actually touching my body she moved the energy around me. It made me feel sick and I nearly fainted, she had to stop doing the reiki and was concerned because she said I looked very pale. I almost got sick with the sensation. **That sensation was the same as the one I had in my dream**. **But as I done many OBE in my dream** the horrible sensation did not feel as bad the more I done. [sic] (OBERF)

# **REFERENCES**

Crookall, R. (1966). The Study and Practice of Astral Projection, University Books.

de Foe, A. (2016). Consciousness Beyond the Body: Evidence and Reflections, Melbourne Centre for Exceptional Human Potential.

DreamJournal. Retrieved 4/10/2023, from <http://www.dreamjournal.net/>.

Father "X", A. C. M. P. (1985). "Lucid Dreams or Out-of-Body Experiences: A Personal Case." Lucidity Letter **4**(2).

Green, C. E. (1968). Lucid Dreams. London, Published for the Institute of Psychological Research by Hamilton.

Green, C. E. (1968). Out-of-the-body Experiences, Institute of Psychophysical Research.

LaBerge, S. (1986). Lucid dreaming. New York, Ballantine Books.

LaBerge, S. and H. Rheingold (1997). Exploring the World of Lucid Dreaming, Ballantine Books.

LDE. "Lucid Dreaming Experience." Lucid Dreaming Experience magazine Retrieved 4/5/2023, from <https://www.luciddreamingmagazine.com/>.

Magallón, L. L. (1997). Mutual Dreaming: When Two Or More People Share the Same Dream, Pocket Books.

Monroe, R. A. (2014). Journeys Out of the Body: The Classic Work on Out-of-Body Experience, Harmony/Rodale.

OBERF. "Out of Body Research Foundation." Retrieved 4/5/2023, from <https://www.oberf.org/index.html>.

Peterson, R. and C. Tart (2013). Out-of-Body Experiences: How to Have Them and What to Expect, Hampton Roads Publishing.

Sellers, J. (2017). "Out-of-Body Experience: Review & a Case Study."

Tholey, P. (1989). "Consciousness and abilities of dream characters observed during lucid dreaming." Percept Mot Skills **68**(2): 567-578.

Van Eeden, F. (1913). "A study of dreams." Proceedings of the Society for Psychical Research **26**.

Waggoner, R. (2008). Lucid Dreaming: Gateway to the Inner Self, Red Wheel Weiser.
